# Qingpeng Ointment Ameliorates Inflammatory Responses and Dysregulation of Itch-Related Molecules for Its Antipruritic Effects in Experimental Allergic Contact Dermatitis

**DOI:** 10.3389/fphar.2019.00354

**Published:** 2019-04-09

**Authors:** Xuan Gong, Hui Xiong, Sisi Liu, Yutong Liu, Liang Yin, Chuyue Tu, Hua Wang, Zhongqiu Zhao, Weiwu Chen, Zhinan Mei

**Affiliations:** ^1^School of Pharmaceutical Sciences, South-Central University for Nationalities, Wuhan, China; ^2^College of Life Science, South-Central University for Nationalities, Wuhan, China; ^3^Center for the Study of Itch, Department of Anesthesiology, Washington University School of Medicine in St. Louis, St. Louis, MO, United States; ^4^Barnes-Jewish Hospital, St. Louis, MO, United States; ^5^Qizheng Tibetan Medicine Co., Ltd., Lanzhou, China

**Keywords:** Tibetan medicine, chronic itch, allergic contact dermatitis, MAPK, mice

## Abstract

The pathogenesis of itchy skin diseases including allergic contact dermatitis (ACD) is complicated and the treatment of chronic itch is a worldwide problem. One traditional Tibetan medicine, Qingpeng ointment (QP), has been used in treatment of ACD in China for years. In this study we used HPLC and LC/MS analysis, combined with a BATMAN-TCM platform, for detailed HPLC fingerprint analysis and network pharmacology of QP, and investigated the anti-inflammatory and antipruritic activities of QP on ACD induced by squaric acid dibutylester (SADBE) in mice. The BATMAN-TCM analysis provided information of effector molecules of the main ingredients of QP, and possible chronic dermatitis-associated molecules and cell signaling pathways by QP. In ACD mice, QP treatment suppressed the scratching behavior induced by SADBE in a dose-dependent manner and inhibited the production of Th1/2 cytokines in serum and spleen. Also, QP treatment reversed the upregulation of mRNAs levels of itch-related genes in the skin (TRPV4, TSLP, GRP, and MrgprA3) and DRGs (TRPV1, TRPA1, GRP, and MrgprA3). Furthermore, QP suppressed the phosphorylation of Erk and p38 in the skin. In all, our work indicated that QP can significantly attenuate the pathological alterations of Th1/2 cytokines and itch-related mediators, and inhibit the phosphorylation of MAPKs to treat the chronic itch.

## Introduction

Chronic itch has been long known not only as a concomitant manifestation of various skin diseases, such as contact dermatitis, eczema, atopic dermatitis, psoriasis, etc., but also occurs in systemic, neurological, or psychiatric diseases. So far, medications for chronic itch still can be simply categorized into topical (emollients and moisturizers, anesthetics, coolants, glucocorticoids, others) and systemic (antihistamines, neuroactive medications, antidepressants, phototherapy, etc.) forms (Yosipovitch and Bernhard, 2013; Pereira and Stander, 2017; Hachisuka et al., 2018; Shevchenko et al., 2018). Due to its heterogeneity, the causes and pathophysiology of chronic itch are complicated. The treatment of chronic itch, especially intractable itch, is a worldwide problem and a great challenge for clinicians.

Complementary and alternative medicine (CAM), including herbal or botanical therapies, traditional Chinese medicine (TCM), dietary supplements, etc., is clinically used to treat intractable skin diseases like psoriasis. A large amount of evidence indicated those CAM managements could effectively relieve the symptoms of diseases or their underlying causes, which cannot be solved by western medicine (Farahnik et al., 2017; Parker et al., 2017; Svendsen et al., 2017; Na Takuathung et al., 2018). For example, it is estimated that there are about 10–62% of patients with psoriasis in the western world seeking the use of CAM (Farahnik et al., 2017; Svendsen et al., 2017). Consistently animal experimental studies have also accumulated a wealth of data demonstrating the effectiveness of the use of CAM in animal models (Na Takuathung et al., 2018).

Allergic contact dermatitis (ACD) is a skin T cell-mediated immune response caused by one or more non-toxic allergens in contact with the skin, and often occurs together with irritant contact dermatitis significantly affecting the quality of life (Kostner et al., 2017). Existing treatments for ACD are still limited to a few traditional methods such as topical steroids and immunosuppressants, etc., etiologically based and efficient treatments are still lacking. Over centuries Chinese medicines have been used in the treatment of ACD, and their main ingredients and possible molecular mechanisms have been gradually recognized (Li and Li, 2013; Li et al., 2013, 2018; Fu et al., 2015). One traditional Tibetan medicine, Qingpeng ointment (QP), has been used in treatment of ACD in China for many years. QP contains nine main ingredients including *Oxytropis falcata* Bunge, *Rheum lhasaense*, *Aconitum pendulum* Busch, *Terminalia chebula* Retz (stoned), *Terminalia billerica* (Gaertn.) Roxb, *Phyllanthus emblica* Linn., *Styrax tonkinensis* (Pierre) Craib ex Hartw, *Tinospora sinensis* (Lour.) Merr., and *Synthetic musk* (Product Manual, Qizheng Tibetan Medicine Co., Ltd., Lanzhou, China). Two previous works reported that QP exerts anti-inflammation and ameliorates ACD in BALB/c mice (Li and Li, 2013; Li et al., 2013, 2018; Fu et al., 2015). However, similar to other TCM experimental studies on ACD they have some common problems. For example, their ACD model was established by topical 4-dinitrofluorobenzene (DNFB), a model that has been used for a long time but still has actual unknown molecule mechanisms (Li and Li, 2013; Li et al., 2013, 2018; Fu et al., 2015). Secondly without exception, they all focus on anti-inflammatory effect and none of them have comprehensively studied the chronic itch associated with ACD.

Squaric acid dibutylester (SADBE), a small molecule hapten, is commonly used in the treatment of alopecia areata and has been used as a popular ACD inducer in mice (Fu et al., 2014; Feng et al., 2017; Luo et al., 2018). Remarkably in recent years neuroimmune mechanisms of SADBE-induced chronic itch were greatly investigated. It was showed that the increased CXCL10/CXCR3 signaling in sensory neurons may contribute to the pathogenesis of the chronic itch in this model (Fu et al., 2014). Most recently we and our collaborators found transient receptor potential (TRP) channels, TRPV1 and TRPA1-expressing sensory neurons or TRPV4 in the TRPV4-expressing epithelial and immune cells in the skin are molecular and cellular basis for SADBE-induced chronic itch (Feng et al., 2017; Luo et al., 2018).

In order to fully understand the pharmacological functions of herbal ingredients or components, we also employed a newly developed bioinformatics analysis tool, BATMAN-TCM (a Bioinformatics Analysis Tool for Molecular mechanism of Traditional Chinese Medicine) established by our collaborator. Unlike the traditional concept of “one drug, one goal,” BATMAN-TCM tries to seek the key molecules targeted by the main components of TCM, and discover the biological networks derived from these molecules (Liu Z. et al., 2016; Zhang et al., 2017). Meanwhile, the data will be compared with the molecular targets and signal pathway networks related to diseases established in the database, and will describe the complicated mechanisms and potential therapeutic values of the drug (Liu Z. et al., 2016; Zhang et al., 2017, 2018). Therefore, this study attempted to introduce BATMANTCM into the ACD study and investigate the anti-inflammatory activities of QP on ACD induced by SADBE in mice, especially whether QP treats the chronic itch, and if it does what the possible mechanism could be.

## Materials and Methods

### QingPeng Ointment Preparation and HPLC Analysis

The QP ointment used in this study was purchased from Tibet Qizheng Tibetan Medicine Co., Ltd. (Lanzhou, China) and the same batch of drug product was sued for all animal experiments, LC/MS analysis and BATMAN-TCM Target Prediction and Analysis. QP ointment contains nine kinds of herbal components, including *O. falcata* Bunge, *R. lhasaense*, *A. pendulum* Busch, *T. chebula* Retz (stoned), *T. Billerica* (Gaertn.)Roxb, *P. emblica* Linn., *S. tonkinensis* (Pierre) Craib ex Hartw, *T. sinensis* (Lour.) Merr., and *S. musk*, which were mixed at a weight ratio of 20:10:15:20:20:20:7:30:5.

For identification of the main constituents, the mass spectral analyses were performed using an Agilent 6540 Q-TOF LC/MS system (Agilent Technologies, Santa Clara, CA, United States). The operating conditions for ESI interface were as following: positive/negative ionization mode; spray voltage, 3.8 kV; gas temperature, 350°C; and the full scan range was from 100 to 1600 *m*/*z*. An Agilent 1290 HPLC system equipped with a G1379A HPLC pump, a G1315B DAD detector and a ZORBAX SB-C18 column (250 × 4.6 mm, 5 μm) was used for the HPLC analysis. The mobile was consisted of acetonitrile (solvent A) and 5% methanol aqueous solution (including 0.1% formic acid, solvent B) and the gradient elution program was as below: 0∼10 min, 100% B; 10∼25 min, 0–5% A, 100∼95% B; 25∼60 min, 5∼10% A, 95∼90% B; 60∼100 min, 10∼20% A, 90∼80% B; 100∼150 min, 20∼70% A, 80∼30% B, 150∼180 min, 70% A, 30% B. The flow rate was 1.0 mL/min, and the sample injection volume was 10 μL. The detection wavelength was set at 284 nm.

### BATMAN-TCM Target Prediction and Analysis

The target prediction method adopted the BATMAN-TCM platform, which in principle, is a similarity-based method to predict potential drug–target interactions based on the similarity of those known TCMs drug–target interactions. Simply, the combination of QP composition and their corresponding PubChem ID were an input into BATMAN-TCM software for network pharmacological analysis to obtain the results of the “drug-disease” potential target prediction (Liu Z. et al., 2016; Zhang et al., 2017). The analysis was based on multiple source database included TCMID database (providing a giant formula-herb-ingredient association data), DrugBank (downloaded on July 26, 2015), KEGG (version: July 31, 2014), TTD (Therapeutic Target Database) (version: 4.3.02), OMIM (Online Mendelian Inheritance in Man) (downloaded on Mar. 13, 2014), and TTD (version: 4.3.02) (Liu Z. et al., 2016; Zhang et al., 2017).

### Animals and Drugs

Male C57BL/6 mice were purchased from Beijing Vital River Laboratory Animal Technology Co., Ltd. (Beijing, China). Animals were single-caged and housed in a SPF-grade environment with water and food *ad libitum* at temperature (24 ± 2)°C, a 12-h/12-h light/dark cycle lighting off at night (7:00 pm–7:00 am), at least adapted for 1 week before behavior work. All behavioral tests were videotaped above the observation boxes, and assessed by observers blinded to the experimental design. In this study, mice used for behavioral tests were divided into the blank control group (acetone topically), the model group (0.5% SADBE/acetone topically), groups of treatment low-dose, medium-dose and high-dose of QP ointment applied to the shaved nape of the neck (0.625, 1.25, and 2.5 g/kg), or the positive drug (Diphenhydramine Hydrochloride, 10 mg/kg, i.p.). In this study, male mice at 7 weeks old were used for experiments. All animal experimental procedures followed international guidelines for care and use of laboratory animals which were approved by the Animal Ethics Committee of South-central University for Nationalities. SADBE was purchased from J&K Scientific Ltd. (Beijing, China). QP was purchased from Tibet Qizheng Tibetan Medicine Co., Ltd. (Lanzhou, China). Diphenhydramine hydrochloride was purchased from Shanghai Yuanye Bio-Technology Co., Ltd. (Shanghai, China).

### Mouse Model of ACD and Treatments

The model was mainly established following previous work (Feng et al., 2017; Luo et al., 2018). The mice were sensitized once per day for three consecutive days (from day 0 to day 2) by applying 20 μL of 0.5% SADBE/acetone solution topically to the shaved abdominal skin. 5 days later, 20 μL of 0.5% SADBE/acetone solution was applied topically to shaved mouse neck skin about 3 × 3 cm region in the midline backs once a day for three consecutive days. 3 days later, the paw scratching behavior was recorded by the camera under unmanned conditions for 60 min, and the number of scratches to the shaved part of the neck was counted by people blinded to the experimental design. The treatment group was topically applied to the shaved nape of the neck with low, medium and high dose QP (0.625, 1.25, and 2.5 g/kg) twice a day starting from day 0 to day 12, and once a day on day 13. Diphenhydramine (10 mg/kg i.p.) as the positive drug was injected into the mice on day 13. The following scratching behavior was recorded after 30 min at the last QP and diphenhydramine treatment by observers. The control group was used same volume of acetone other than 0.5% SADBE/acetone. The model group mice were applied to the blank substrate (from Tibet Qizheng Tibetan Medicine Co., Ltd.) at the same time (Feng et al., 2017; Luo et al., 2018).

### Skin Morphology Studies

The skin in midline of the mouse neck was collected and fixed in 4% paraformaldehyde, routinely dehydrated, embedded in paraffin, and sectioned. Hematoxylin & eosin (HE) and toluidine blue staining were performed as previously described (Zhao et al., 2013). After the stained sections were dehydrated, cleared and sealed, they were visualized under a microscope, and images were taken. Image J software was used to measure the relative thickness of the epidermis from HE staining images and count the number of mast cells from toluidine blue staining images.

### SDS–PAGE and Western Blotting

The total protein from the mouse midline back neck skin and cervical Dorsal Root Ganglion (DRG) (C1–C7) were extracted as according to standard protocols. Simply, a 1% protease inhibitor (Roche, Switzerland) and a 1% phosphatase inhibitor (Roche, Switzerland) were added to the protein lysate. By using this lysate buffer, the mouse tissues were homogenized and sonicated, then placed on ice for 30 min, and centrifuged (12000 *g*, 4°C, 9 min) to take the supernatants for the next steps. Samples were denatured in a 20% 5× loading buffer and heated in a boiling water bath for 8 min. After cooling, the samples were separated and cryopreservation. Proteins were separated by SDS–PAGE (7 or 10% acrylamide) and blotted onto PVDF membranes. The membrane was incubated with primary antibodies overnight at 4°C, and the primary antibodies involved were anti-P38 (8690S, 1:1000), anti-phospho-P38 (4511S, 1:1000), anti-Erk (4695S, 1:1000), anti-phospho-Erk (4370P, 1:2000), anti-Akt (4691S, 1:1000), anti-phospho-Akt (4060S, 1:2000), anti-Stat3 (12640S, 1:1000), anti-phospho-Stat3 (9145P, 1:2000), anti-IκBα (4812S, 1:1000), anti-phospho-IκBα (2859S, 1:2000) (CST, Danvers, MA, United States) and anti-Tubulin (Proteintech, Wuhan, China, 1:10000). Then the membranes were incubated with the appropriate horseradish peroxidase-conjugated secondary antibodies for 1 h at room temperature. The following immunoreactive polypeptides were visualized using ECL (ZETA, San Francisco, CA, United States) assays, and Tubulin was used as an internal control.

### Quantitative RT-PCR

Total RNA was extracted from homogenized DRG neurons or skin samples using the Trizol reagent (TaKaRa, Japan) according to the manufacturer’s instructions. RNA was treated with DNase I and cDNA was synthesized *in vitro* using a ThermoScript RT-PCR reverse transcription kit. Each reaction was performed in a system containing 10 μL SYBR Mix, 2 μL cDNA, 1 μL primer mix and 7 μL RNase-free water, using a real-time PCR system. Relative mRNA expression levels of different target genes compared to GAPDH or β-Actin were calculated using the 2^−ΔΔCt^ method. The primer sequences (5′ to 3′) involved were showed in Table 1.

**Table 1:** Primer sequences.

Gene name	Forward (5′-3′)	Reverse (5′-3′)
TRPV1	CCCGGAAGACAGATAGCCTGA	TTCAATGGCAATGTGTAATGCTG
TRPV4	CGCCCGCGTCCTGAG	TCCCCCTCAAACAGATTGGC
TRPA1	GCTGGACTGCTTTGCATCAC	AAGCATTGCAACAGCCTTGG
GRP	CCTAGAAGCTGCTGGGAACC	CCCTTGTCGTTGTCCCTTCA
TSLP	CGCCCTCGACTCGGAC	TTCCAGAAGAGCCATCGCAG
MrgprA3	TCCAGCAAGAGGAATGGGAG	CATTGTCGAGGCTGAGGTGT
IL-4	TCTCGAATGTACCAGGAGCCATATC	AGCACCTTGGAAGCCCTACAGA
IL-5	GCTTCTGCACTTGAGTGTTCTG	CCTCATCGTCTCATTGCTTGTC
IL-10	CAAGGAGCATTTGAATTCCC	GGCCTTGTAGACACCTTGGTC
GAPDH	AAGAGGGATGCTGCCCTTAC	ATGAAGGGGTCGTTGATGGC
β-Actin	GTGACGTTGACATCCGTAAAGA	GTAACAGTCCGCCTAGAAGCAC

### Measurement of Cytokines

Detection of IgE, Histamine, TNF-α, IFN-γ, IL-1β, IL-4, IL-5, IL-10, etc., in the spleen and serum of mice using an ELISA kit according to the manufacturer’s instructions. All mouse ELISA kits were purchased from Shanghai Jianglin Biotechnology Co., Ltd.

### Statistical Analysis

All data was expressed as the means ± SEM. GraphPad Prism 6 software (San Diego, CA, United States) was used for the data statistical analysis and graphics. Unpaired *t*-test was used to analyze statistical comparisons between two groups. Multiple comparisons were compared by one-way analysis of variance (ANOVA) followed by Bonferroni’s *post hoc* tests. *p*-value < 0.05 was assumed as statistically significant.

## Results

### HPLC Profile of QingPeng Ointment

**FIGURE 1:**
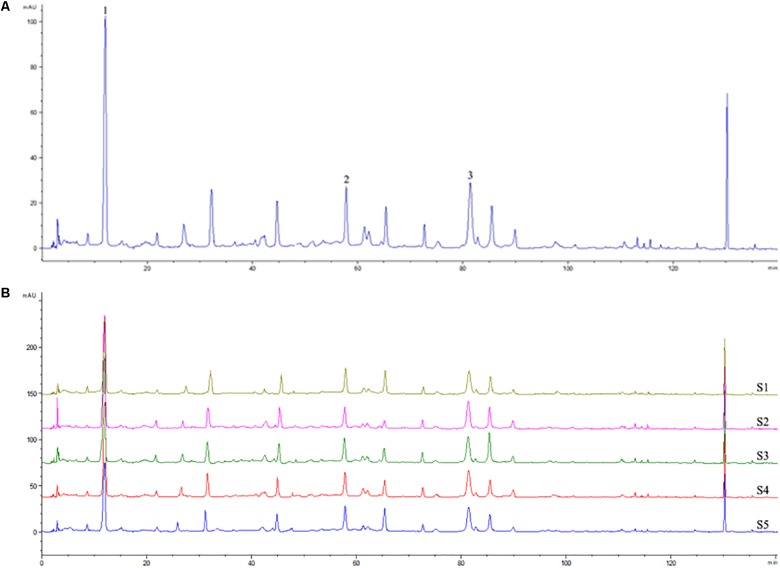
HPLC fingerprint analysis of QP ointment. The HPLC chromatographic of QP at 284 nm wavelength was recorded **(A)**. There are at least 3 phenotypic peaks, which are derived from gallic acid (1), corilagin (2), and ellagic acid (3), respectively. HPLC fingerprint of 5 batches of QP was also presented **(B)**.

Using the current LC/MS method, three main compounds of QP were identified, including gallic acid (1) (Teodoro et al., 2018), corilagin (2) (Salih et al., 2017), and ellagic acid (3) (BenSaad et al., 2017) (their structures showed in Supplementary Figure S1). These compounds are all phenolic acid derivatives and gallic acid as the main unit. In their mass spectrum, fragment ions at *m/z* 465 [M-169]^+^ in the 2 indicated the existence of gallic acid unit, and *m/z* 153, 121 in the 3 indicated the existence of 3,4-dihydroxybenzoic acid and benzoic acid units (Supplementary Figure S1). Five batches purchased from Tibet Qizheng Tibetan Medicine Co., Ltd. were also examined based on the results of LC/MS. These batch numbers were as follows: 161130 (S1), 170308 (S2), 170812 (S3), 171134 (S4), and 180513 (S5). The retention times of three compounds were 12.03, 57.82, and 81.41 min, respectively (Figure 1A,B).

### The Visualization of QP Ingredient-Target-Pathway/Chronic Dermatitis Association Network

**FIGURE 2:**
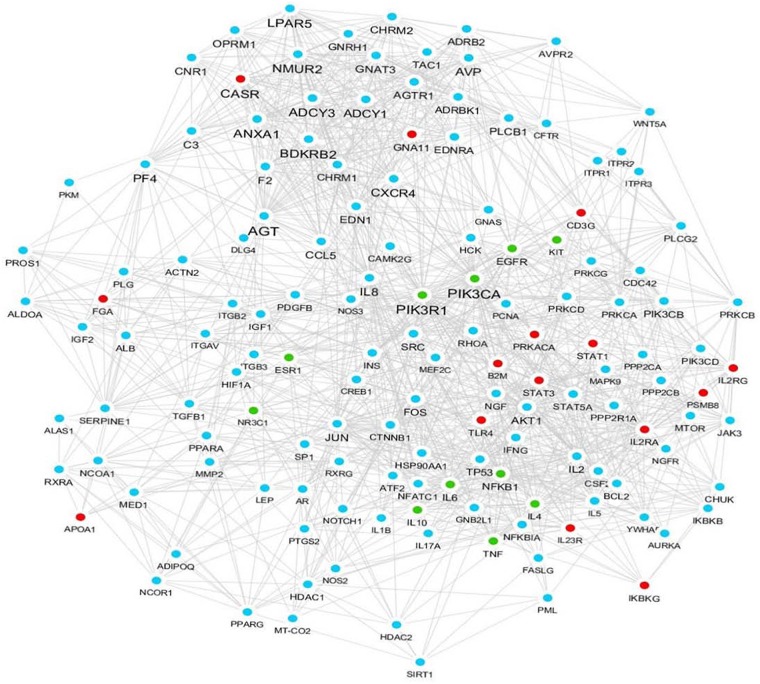
QP ingredient-target-pathway/chronic dermatitis association network. Red nodes correspond to the predicted key targets of chronic dermatitis, such as TLR4, STAT1, STAT3, PRKACA, PSMB8, IL2RG, CD3G, etc. Blue nodes correspond to the key targets of QP ointment, such as IL-8, CXCR4, JUN, IL-1β, IL-17A, etc., Green nodes represent the key targets associate to both QP and chronic dermatitis, such as IL-4, IL-6, IL-10, TNF, NFκB1, PIK3R3, PIK3CA, EGFR, KIT, ESR1, NR3C1, etc.

To establish the related target prediction and network/pathway analysis for QP to treat chronic dermatitis, the current work used the platform of BATMAN-TCM and obtained the interactive network diagram of QP-chronic dermatitis (Figure 2). In the view of the association network, based on QP’s drug targets and biological pathways, targets of chronic dermatitis diseases and predicted pathways, the prediction results are divided into three types of nodes including composition-targets association, disease-targets association and their overlapping association. A complete gene names of targets and biological pathways for QP’s drug and/or chronic dermatitis, see MS Excel worksheets 1 and 2 in Supplementary Table S1.

### QP Attenuated Scratching Behavior Induced by SADBE in Mice

**FIGURE 3:**
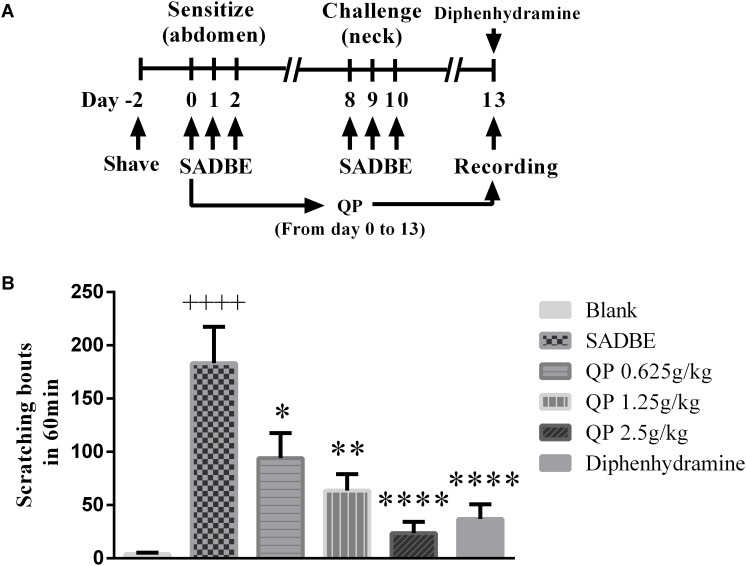
QP reduced the itching caused by allergic contact dermatitis induced by SADBE. **(A)** A Schematic diagram shows the experimental protocol. 0.5% SADBE/acetone was topically treated daily 3 times to the mouse abdomen, and re-stimulated topically daily 3 times to the neck back skin after a 5 days’ break. Spontaneous scratching was videotaped and analyzed afterward. **(B)** The behavior results of SADBE mice in different controls or after three QP (0.625, 1.25, and 2.5 g/kg), and the positive drug (diphenhydramine, 10 mg/kg). Data was expressed as mean ± SEM. ^∗^*p* < 0.05, ^∗∗^*p* < 0.01, and ^∗∗∗∗^*p* < 0.0001, compared with the model group. ^++++^*p* < 0.0001, compared with the blank control group. One-way ANOVA followed by *post hoc* test (*n* = 5 each group). The method and criteria to present and statistically analyze the data in **(B)** were followed the same in all other work in this paper unless mentioned specifically.

Skin allergies and chronic dermatitis have been caused by topical application of SADBE, a popular ACD inducer in mice (Fu et al., 2014; Feng et al., 2017; Luo et al., 2018). In this study, we followed the schematic experimental protocol (Fu et al., 2014; Feng et al., 2017; Luo et al., 2018) and pretreated mice with SADBE in the abdomen for 3 days (sensitization) and re-stimulated after 3 days topically on the neck (challenge), which induced robust spontaneous scratching behavior in mice (Figure 3A). Previous analyses indicated that no corticosteroid existed in QP (Li and Li, 2013; Li et al., 2013) and antihistamines have been used traditionally in the attenuation of experimental and clinical pruritus (Yosipovitch and Bernhard, 2013; Mahapatra et al., 2014; Pereira and Stander, 2017; Hachisuka et al., 2018; Shevchenko et al., 2018), therefore in this study we used a classic antihistamine, diphenhydramine, as the positive control medicine. Similar to previous reports (Fu et al., 2014; Feng et al., 2017; Luo et al., 2018), compared with the blank control mice, SADBE indeed induced robust scratching behaviors (*p* < 0.0001) (Figure 3B). All three QP treatments (0.625, 1.25, and 2.5 g/kg) and the positive drug (diphenhydramine, 10 mg/kg) significantly decreased the scratching behavior than that in the model group (*p* < 0.05). It is worth noting that QP can work in a dose-dependent manner to suppress the pruritus response in the ACD model (Figure 3B).

### Effect of QP on Skin Histological Changes Induced by SADBE

**FIGURE 4:**
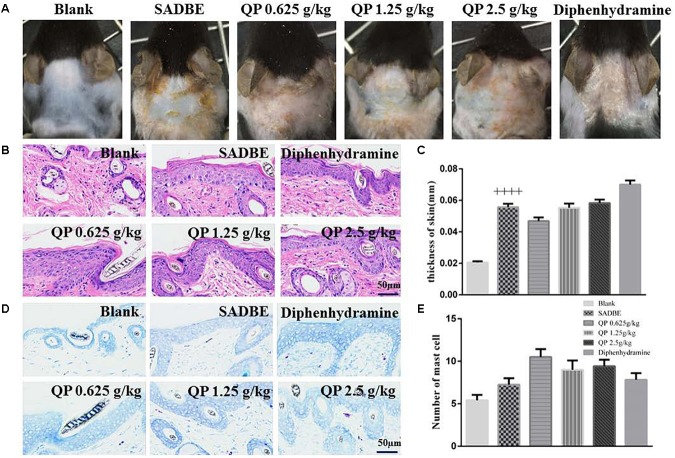
Effect of QP on skin histological changes in SADBE mice. **(A)** The midline dorsal neck skin images of each group of mice were showed on day 13. The neck skin lesions in QP treatment groups were fewer than those in the model group. **(B,C)** showed representative HE staining images and measured skin thickness of each group as labeled, respectively. **(D,E)** showed representative toluidine blue images and cell counts of each group as labeled, respectively. Data was expressed as mean ± SEM. ++++*p* < 0.0001, compared to the blank group. One-way ANOVA (*n* = 5 each group). Scale bar: 50 μm.

We next examined the SADBE induced skin lesions and whether the QP treatments were beneficial to them. From the skin general appearance observed, compared with the model group, mice after treated with different doses of QP showed some signs of reduced skin lesions (Figure 4A). However, from the results of HE (Figure 4B,C) and toluidine blue (Figure 4D,E) staining, there was no significant differences after any QP or diphenhydramine treatment as compared with the model group, and by the measurement of skin thickness and the number of mast cells, which have been frequently used as indicators for the skin inflammation and immune cell infiltration. The data indicated, in mice SADBE initiated thickening of skin and increased infiltration of mast cells, which could not be significantly inhibited by QP.

### QP Inhibited Upregulation of Cytokines and Itch Molecules in the Skin of SADBE Mice

**FIGURE 5:**
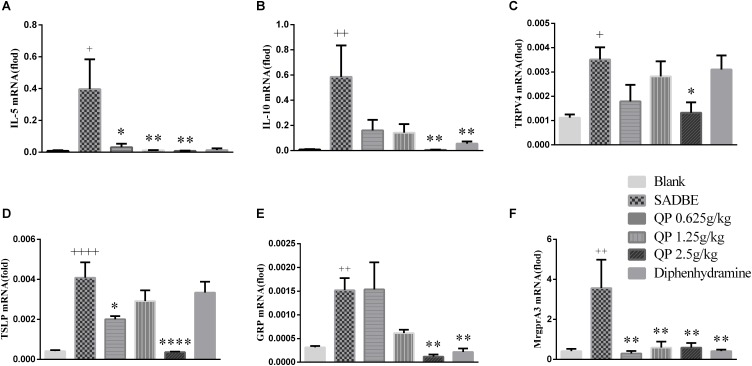
The mRNA of IL-5 **(A)**, IL-10 **(B)**, TRPV4 **(C)**, TSLP **(D)**, GRP **(E)**, and MrgprA3 **(F)** were detected in mouse skin. After mice were treated with different doses of QP ointment and diphenhydramine, the targeted molecular mRNA levels were measured as well as that in the model group or the blank control group. Data was expressed as mean ± SEM. ^∗^*p* < 0.05, ^∗∗^*p* < 0.01, and ^∗∗∗∗^*p* < 0.0001, compared with the model group. ^+^*p* < 0.05, ^++^*p* < 0.01, and ^++++^*p* < 0.0001, compared with the blank control group. One-way ANOVA (*n* = 5 each group).

In this study we tried to take advantage of the information from our network pharmacological study (Figure 2 and Supplementary Table S1) for molecular analysis. Upon the results of potential effector molecules they provided, we analyzed the mRNA expression levels of IL-5, IL-10, TRPV4, TSLP, GRP, and MrgprA3 in the skin from different groups of mice. The experimental results showed that the mRNA levels of IL-5, IL-10, TRPV4, TSLP, GRP, and MrgprA3 in the skin of the model group were significantly higher than those in the blank control group. Compared with the model group, QP treatments (especially at the high dose group) can significantly reduce the enhanced IL-5, IL-10, TRPV4, TSLP, GRP, and MrgprA3 mRNA levels in the skin of SADBE mice (Figure 5). The positive control drug diphenhydramine, was able to significantly decrease that of IL-10, GRP, and MrgprA3.

### QP Inhibited p-P38 and p-Erk Signaling Augments in the Skin of SADBE Mice

**FIGURE 6:**
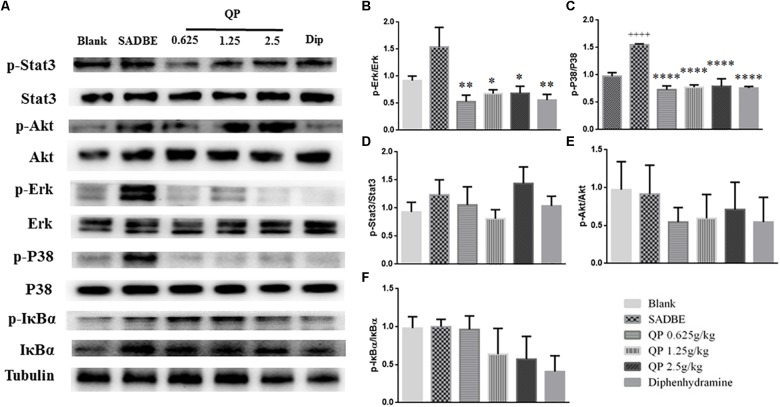
QP suppressed the phosphorylation of MAPKs in the skin of SADBE mice. Representative western blots images of p-Stats, Stats, p-Akt, Akt, p-Erk, Erk, p-P38, p38, p-IκBα, IκBα, and Tubulin, respectively **(A)**. Expression of p-Erk **(B)** and p-P38 **(C)** but not p-Stat3 **(D)**, p-Akt **(E)**, and p-IκBα **(F)** in the skin of SADBE mice was significantly reduced QP compared to that in the model group. Data was expressed as mean ± SEM. ^∗^*p* < 0.05, ^∗∗^*p* < 0.01, and ^∗∗∗∗^*p* < 0.0001, compared to the model group. ^++++^*p* < 0.0001, compared to the blank control group. ANOVA (*n* = 3 each group). The related original western blot images were showed in Supplementary Figures S3, S4.

It is well known a number of intracellular kinase cascades would be activated by inflammatory mediators and participate in the generation and maintenance of immune responses in dermatitis (Ayush et al., 2013; Bechara et al., 2017; Oetjen et al., 2017; Li et al., 2018; Xiao and Xiao, 2018). Considering the information from our network pharmacological study (Figure 2 and Supplementary Table S1), expression of several important protein kinases was detected in the skin of mice (Figure 6A–F). Compared with the blank control group, the level of phosphorylated P38 (Figure 6C) in the skin of the model group increased significantly. Compared with the model group, the high-dose QP significantly decreased the expression of p-P38 and p-Erk (Figure 6B,C) in the skin. Detection of the p-Stat3, p-Akt and p-IκBα expression in the skin of mice revealed that there was no significant difference in their expression levels among the groups (Figure 6A,D–F).

### QP Inhibited Upregulation of Cytokines and Itch Molecules in the DRGs of SADBE Mice

**FIGURE 7:**
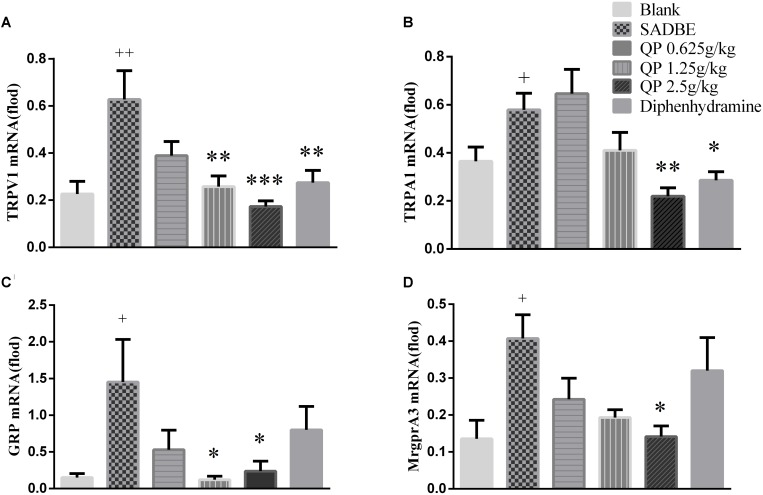
The mRNA of TRPV1 **(A)**, TRPA1 **(B)**, GRP **(C)** and MrgprA3 **(D)** were detected in mice DRG. After mice were treated with different doses of QP ointment and diphenhydramine, the targeted molecular mRNA levels were measured as well as that in the model group or the blank control group. Data was expressed as mean ± SEM. ^∗^*p* < 0.05, ^∗∗^*p* < 0.01, and ^∗∗∗^*p* < 0.001, compared to the model group. ^+^*p* < 0.05 and ^++^*p* < 0.01, compared to the blank control group. ANOVA (*n* = 5 each group).

The DRG is the site where the primary neuronal cells are clustered and also a critical site for the development itch sensory information in the periphery (Han et al., 2013; Liu Z. et al., 2016; Kittaka and Tominaga, 2017; Zhang et al., 2017). Real-time PCR was used to measure the mRNA levels of TRPV1 (Figure 7A), TRPA1 (Figure 7B), GRP (Figure 7C), and MrgprA3 (Figure 7D) in the DRG of mice. As shown in Figure 7, the levels of TRPV1, GRP, and MrgprA3 mRNA in the DRG of model group were significantly higher than those in the blank control group. The positive drug diphenhydramine was effective in reducing TRPA1 and TRPV1 mRNA levels. Even though the low-dose QP had no significant effect on the four itch-related genes, however, compared with that of the model group, TRPV1 and GRP mRNA levels were significantly reduced by the medium-dose of QP, and TRPV1, TRPA1, GRP, and MrgprA3 mRNA levels were markedly reduced by the high-dose of QP.

### QP Altered the Levels of Cytokines and IgE in the Serum or Spleen in SADBE Mice

**FIGURE 8:**
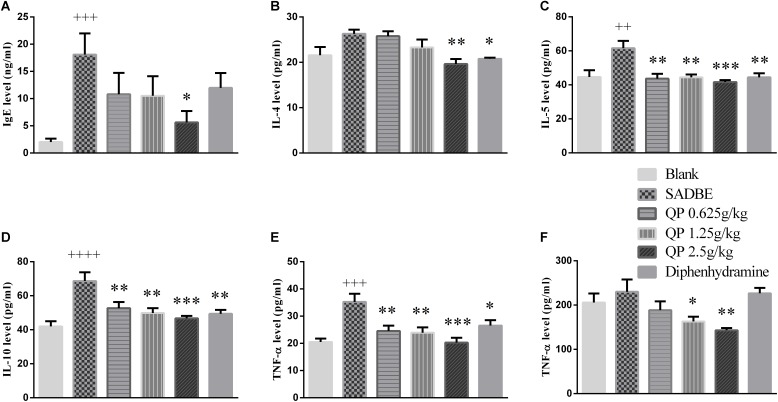
Effect of QP on the levels of different cytokines or IgE in the serum or spleen of SADBE mice. Among them, **(A–E)** showed the levels of IgE, IL-4, IL-5, IL-10, and TNF-α in the serum of each group, and **(F)** showed of TNF-α in the spleen of each group. ^∗^*p* < 0.05, ^∗∗^*p* < 0.01, and ^∗∗∗^*p* < 0.001, compared to the model group. ^++^*p* < 0.01, ^+++^*p* < 0.001, and ^++++^*p* < 0.0001, compared to the blank control group. ANOVA (*n* = 5 each group).

By using the IgE, IL-4, IL-5, IL-10, and TNF-α Elisa kits, the levels of IgE (Figure 8A), IL-4 (Figure 8B), IL-5 (Figure 8C), IL-10 (Figure 8D), TNF-α (Figure 8E) in the serum and TNF-α (Figure 8F) in the spleen were detected. Compared with that in the blank control mice, the production of IgE, IL-5, IL-10 and TNF-α in the model group mice were significantly increased. Positive control drug could significantly reduce IL-4, IL-5, and IL-10 and TNF-α content in serum, but QP showed good inhibitory effect in reducing the content of all these cytokines in the serum or spleen. Among them, the high-dose of QP showed the most striking effect which significantly to all markers detected.

## Discussion

Recent advances in experimental and computational studies have shown that, unlike some single drugs of western medicine, natural medicines or TCM mostly contain multiple components and exert their therapeutic effects by acting on multiple targets (Liu Z. et al., 2016; Zhang et al., 2017, 2018). Therefore, it is necessary to introduce modern technologies and systematic methods, such as HPLC fingerprint analysis and network pharmacology, to reveal the pharmacological functions of these drugs and predict their action mechanisms (Liu Z. et al., 2016; Zhang et al., 2017, 2018). In this study the HPLC analysis was used to characterize the phytochemical features of QP ointment and 3 common peaks were proposed as the fingerprints of its multiple chemical constituents. According to the fragment ion at *m/z* 153 in the mass spectrum indicated that QP contained major phenolic constituents with the central structural unit of gallic acid. Previously this type compounds had been showed treatment effects on ACD or contact dermatitis (Mo et al., 2014; Fu et al., 2015). However, even though gallic acid, corilagin, and ellagic acid were identified as major components of QP, there may be other effective or novel structural compounds in QP since there are several peaks of HPLC fingerprint analysis. Further investigation is needed to investigate what they are and whether they are effective ingredients for the treatment of ACD.

On the other hand, BATMAN-TCM is a bioinformatics analysis tool developed by our collaborators (Liu Z. et al., 2016; Zhang et al., 2017). In this study by using BATMAN-TCM, we analyzed the potential targets of each component of QP, and generated a current ACD’s disease-related gene database. Our results suggested that molecules such as IL-8, CXCR4, JUN, IL-1β, IL-17A, etc., are potential effector molecules or downstream molecules of QP, and molecules such as TLR4, STAT1, STAT3, PRKACA, etc., are chronic dermatitis-associated targets, while IL-4, IL-6, IL-10, TNF, NFκB1, etc., may cover by both. As far as we know, this study is the first use of modern methods of network pharmacology to explore the therapeutic effect of Tibetan medicine on allergic contact dermatitis. Certainly, this method still has some limitations. For example, it mainly relies on the continuous updating and improvement of the relevant online database (Liu Z. et al., 2016; Zhang et al., 2017). The results given by BATMAN-TCM were suggested to be combined with the existing literature and research progress, and the results of animal and cytological experiments were to make the most appropriate judgment. The mRNA-seq technology can provide a method for rapidly distinguishing altered mRNA after drug treatment (Duan et al., 2019), if possible, the mRNA-seq work for studying transcriptome expression in a QP-treated ACD mouse model will help to understand and explain the mechanism of action of QP.

Our experimental results highlighted the key role of several Th1/2 cytokines for QP to treat the chronic itch. T helper (Th) cells have been defined into different subsets by different cytokine profiles secreted. For example, Th1 cells are characterized by the secretion of IL-2, IFN-γ and TNF-α, etc., while Th2 cells secrete IL-4, IL-5, and IL-13, etc (Raphael et al., 2015). Th1 cytokines, like TNF-α/TNFR1 signaling, were showed in animal behavior as required for both acute and chronic itch (Miao et al., 2018). More strikingly Th2 cytokines have been reported as highly involving in the inflammation response directly mediating chronic itch. For example, recent data suggest IL-4, IL-31, and IL-33 all have their own receptors expressing in both mouse and human sensory neurons, and these cytokines enhance neuronal responsiveness to multiple pruritogens (Cevikbas et al., 2014; Kaesler et al., 2014; Liu B. et al., 2016; Oetjen et al., 2017). Our data indicate that high level of interleukin IL-5 was detected in ACD mouse skin, and significantly increased IgE, IL-4, IL-5, IL-10 levels in serum, and as wells as elevated TNF-α in both serum and spleen. On the other hand, QP can significantly decrease the upregulation of these molecules in the serum, spleen and skin. In all, these data would not only support some recent chronic itch progress related to our studies (Zhao et al., 2013; Cevikbas et al., 2014; Kaesler et al., 2014; Liu B. et al., 2016; Oetjen et al., 2017), but also indicated QP can play anti-inflammatory and antipruritic actions through these molecules in multiple places (serum, spleen, skin, and DRG).

Over the past 10 years, effects of various itch mediators expressed by primary sensory neurons and skin cells (like keratinocytes) have been extensively studied. For example, keratinocytes released thymic stromal lymphopoietin (TSLP) could activate sensory neurons and immune cells for itch (Wilson et al., 2013). Several itch specific neuropeptides, like GRP (Sun and Chen, 2007), somatostatin (Huang et al., 2018), and specific neurons like MrgprA3^+^ (Han et al., 2013) and GRPR^+^ (Sun et al., 2009) cells have been identified for the itch signal transmission. TRP channels, especially thermosensitive TRP channels including TRPV1, A1,V4 etc., receive multimodal stimuli and required for itch sensations (Liu Z. et al., 2016; Kittaka and Tominaga, 2017; Zhang et al., 2017). Research studies including our work proved both TRPA1 and TRPV1 channels are required for generating spontaneous scratching induced by SADBE and TRPV4-expressing epithelial and immune cells in the skin dynamically mediate chronic itch (Liu Z. et al., 2016; Zhang et al., 2017). In this study, our work demonstrated the mRNA of TRPV1, TRPA1, GRP, and MrgprA3 were elevated in the SADBE mice DRG and /or skin, which were significantly decreased by QP to a different extent.

Signal transduction originating from membrane-bound Th1/2 cytokine receptors is a complex network that leads to intracellular gene expression and ultimately regulates cellular activities. Among them, the mitogen activated protein kinase (MAPK) (Ayush et al., 2013; Li et al., 2018; Xiao and Xiao, 2018), the nuclear factor kappa-light-chain-enhancer of activated B cells (NF-κB) (Xiao and Xiao, 2018), the Janus kinase (JAK)/signal transducer and activator of transcription (STAT) (Bechara et al., 2017; Oetjen et al., 2017) have been intensively studied and strongly implicated with the pathogenesis of skin diseases and inflammation-controlling interventions. Our work supported, probably QP works through p-P38 and p-Erk cell signaling pathways in skin (not DRG, see Supplementary Figure S2, S5) to treat the chronic itch in ACD mice.

The pathogenesis of chronic skin diseases including ACD is complicated, including at least skin barrier dysfunctions, allergy/immunity, and pruritus (Liu Z. et al., 2016; Kittaka and Tominaga, 2017; Kostner et al., 2017; Zhang et al., 2017; Hachisuka et al., 2018). Cytokines can be produced by a wide array of immune cells, including neutrophils, eosinophils, mast cells, macrophages, and T cells (Liu Z. et al., 2016; Kittaka and Tominaga, 2017; Kostner et al., 2017; Zhang et al., 2017; Hachisuka et al., 2018). Therefore, even though we have some understanding about the possible cytokines, itch-related molecules, and related intracellular signaling pathways for QP to work with, the specific subsets of the cell types, what exact ingredients, and how they function through these cells and molecular network systems, etc., need to be further clarified. Future research on experiments *in vitro*, genetic engineering animals, etc., may help address these issues.

## Conclusion

Via a HPLC system and a BATMAN-TCM platform, for the first time we performed detailed HPLC fingerprint analysis and network pharmacology studies of QP ointment. By in-depth animal and molecular analysis, we identified a number of chronic dermatitis-associated Th1/2 cytokines, itch mediators, and cellular signaling pathways in the skin, DRG and serum for QP to treat the inflammation and chronic itch associated with ACD. Our data clearly suggested, upon stimulation, cytokines and itch-related mediators are released from the skin and neuronal tissues, and QP could effectively work on both the skin and sensory neurons to treat the chronic itch.

## Ethics Statement

All animal experimental procedures followed international guidelines for care and use of laboratory animals which were approved by the Animal Ethics Committee of South-central University for Nationalities.

## Author Contributions

ZM designed and supervised the study. XG, HX, ZZ, and ZM analyzed the data and wrote the manuscript. XG and HX mainly performed the experiments. LY, SL, CT, HW, YL, and WC helped the experiments.

## Conflict of Interest Statement

WC was employed by the company Tibet Cheezheng Tibetan Medicine Co., Ltd. The remaining authors declare that the research was conducted in the absence of any commercial or financial relationships that could be construed as a potential conflict of interest.

## Supplementary Material

DATA SHEET S1 Three main compounds of QP (Figure S1). QP suppressed the phosphorylation of MAPKs in the DRG of SADBE mice (Supplementary Figure S2). The data of the original western blot images (Supplementary Figures S3–S5).

TABLE S1 The key targets of chronic dermatitis or QP (Sheet 1), and targets corresponding to key signaling pathways (Sheet 2) based on the platform of BATMAN-TCM.
