# Supplementary material for: Qingpeng Ointment Ameliorates Inflammatory Responses and Dysregulation of Itch-Related Molecules for Its Antipruritic Effects in Experimental Allergic Contact Dermatitis
Source: Front Pharmacol. 2019 Apr 9;10:354. doi: 10.3389/fphar.2019.00354 (PMC6465648; doi:10.3389/fphar.2019.00354)
Supplement: DATA SHEET S1 — Three main compounds of QP (Figure S1). QP suppressed the phosphorylation of MAPKs in the DRG of SADBE mice (Supplementary Figure S2). The data of the original western blot images (Supplementary Figures S3–S5). [file Data_Sheet_1.docx]

**Supplementary data (Figure S1- Figure S5)**

**A**


**B**


**C**


**D**

**Figure S1∣**Three main compounds of QP.

(**A**) the LC/MS spectrum of QP; (**B**-**D**) the mass spectrums of compound (1-3). gallic acid (**B**), *m/s*:170 and t*_R_* 14.88; corilagin (**C**), *m/s*:634 and t*_R_* 66.87; and ellagic acid (**D**), *m/s*: 302 and t*_R_* 86.11


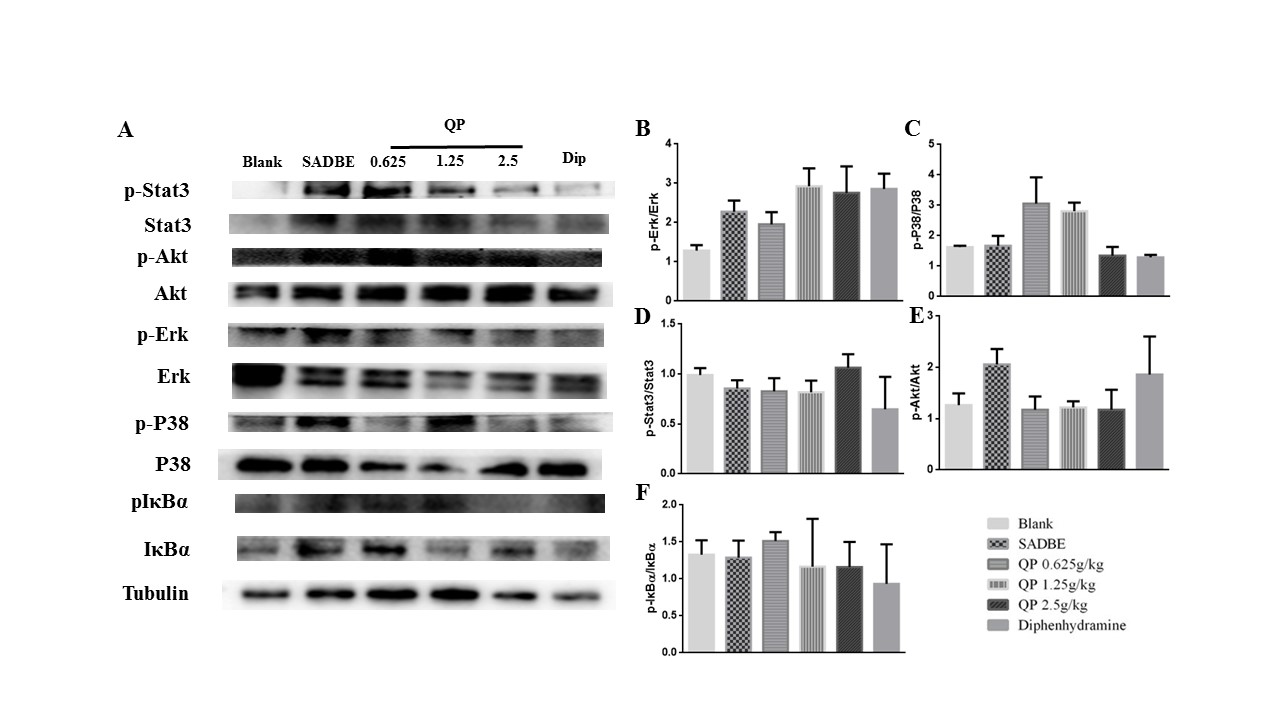


**Figure S2∣**QP suppressed the phosphorylation of MAPKs in the DRG of SADBE mice. Expression of p-Erk, p-P38, p-Stat3, p-Akt, and p-IκBα in the DRG of SADBE mice was not significantly reduced QP compared to that in the model group (*p* > 0.05). Data was expressed as mean ± SEM. ANOVA (n=3 each group).

p-Stat3

**Blank**

**SADBE**

**0.625**

**1.25**

**2.5**

**Dip**

**QP**

**Blank**

**SADBE**

**0.625**

**1.25**

**2.5**

**Dip**

**QP**


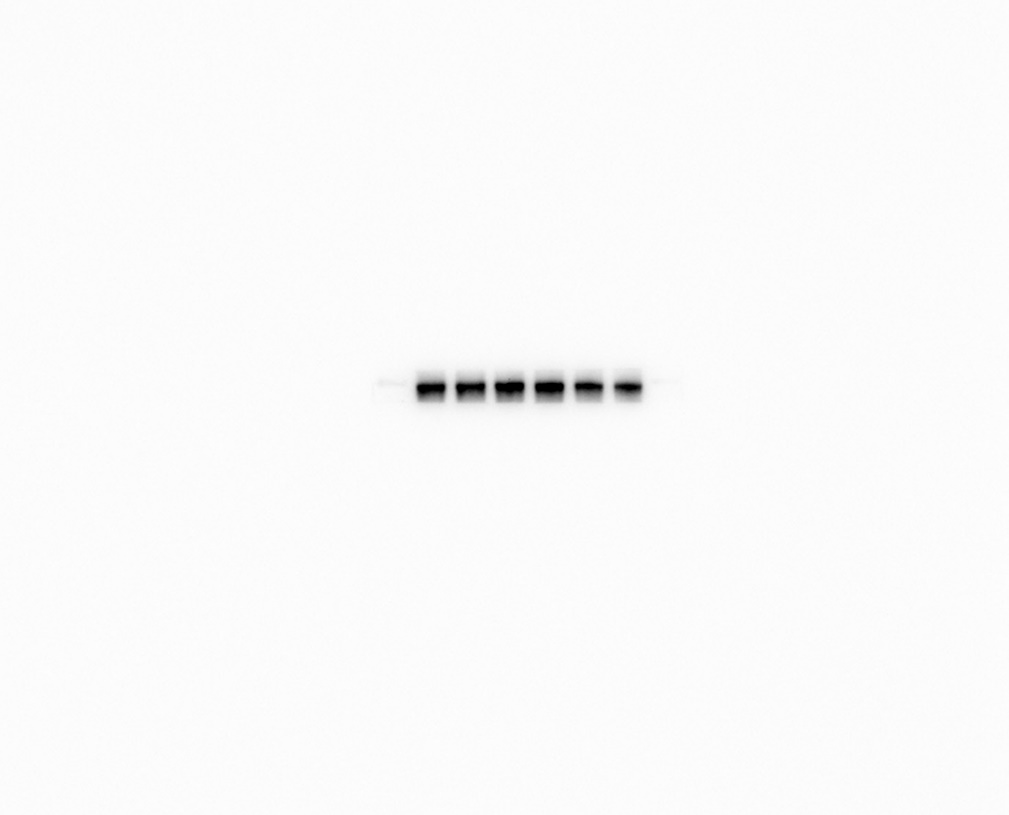

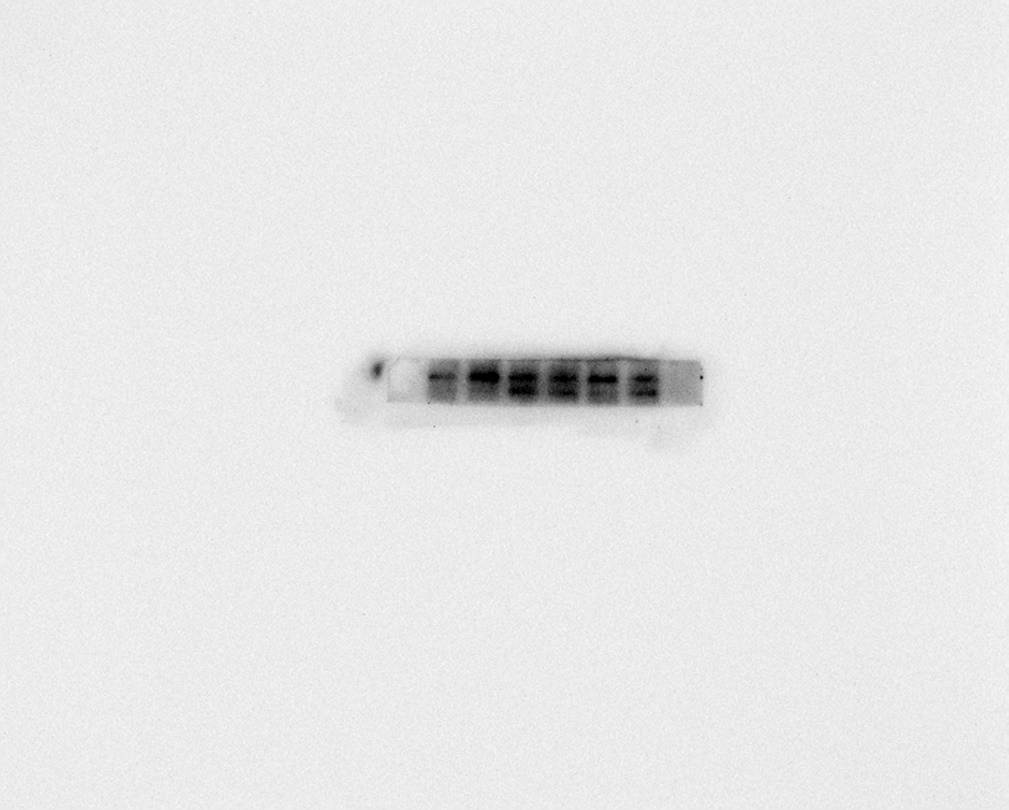

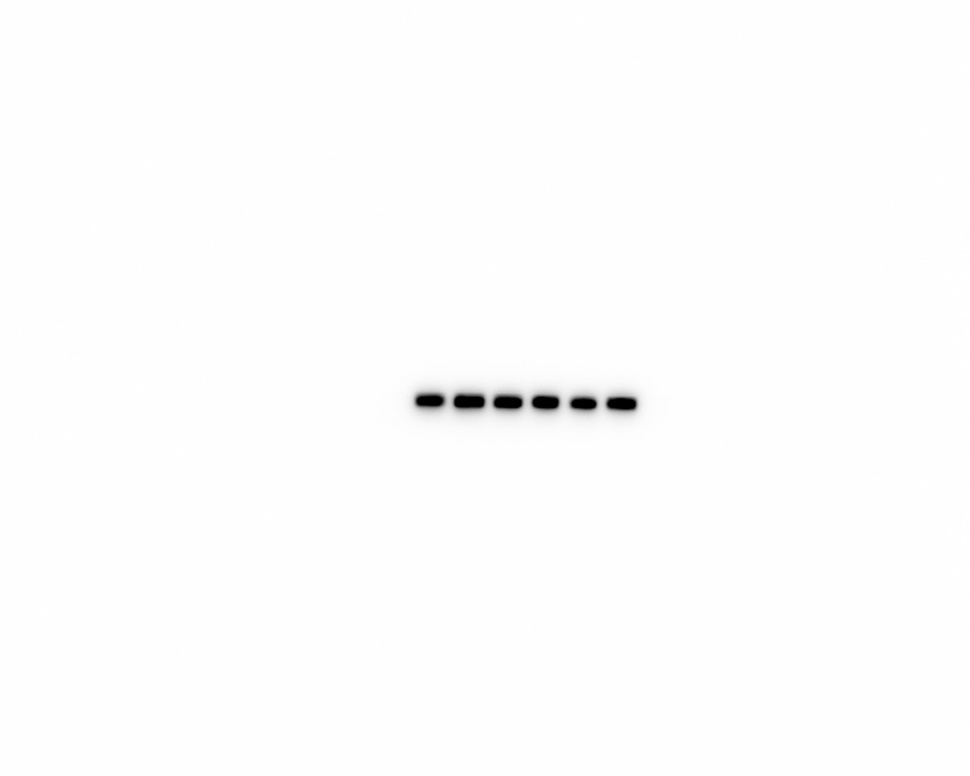

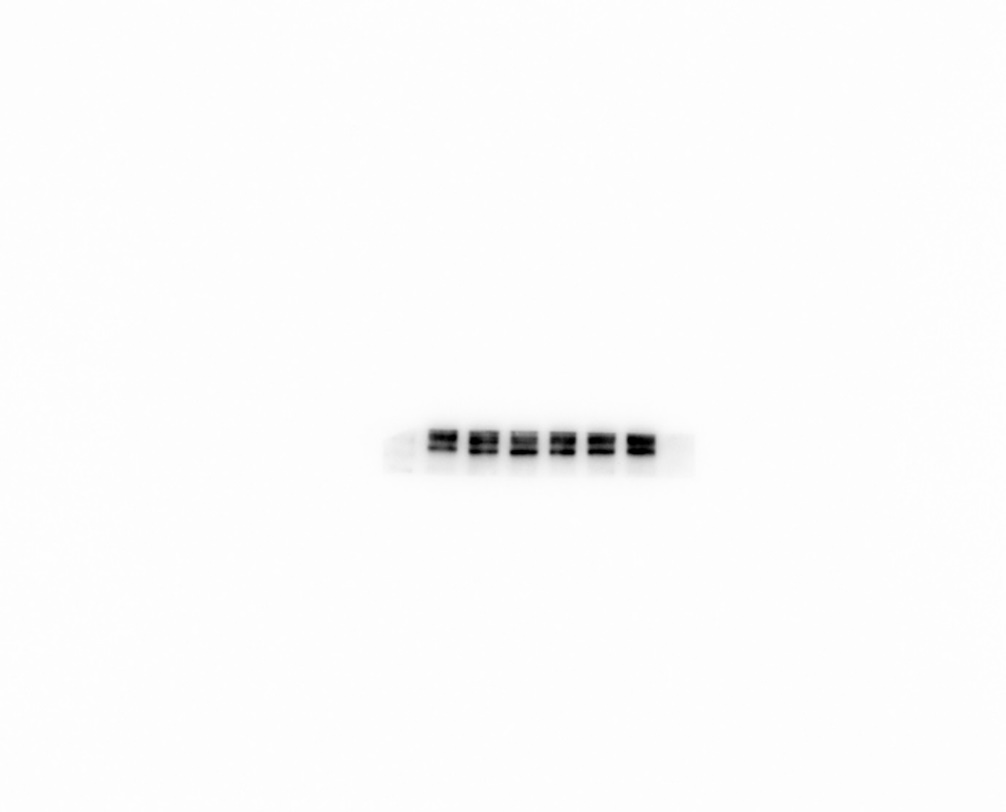

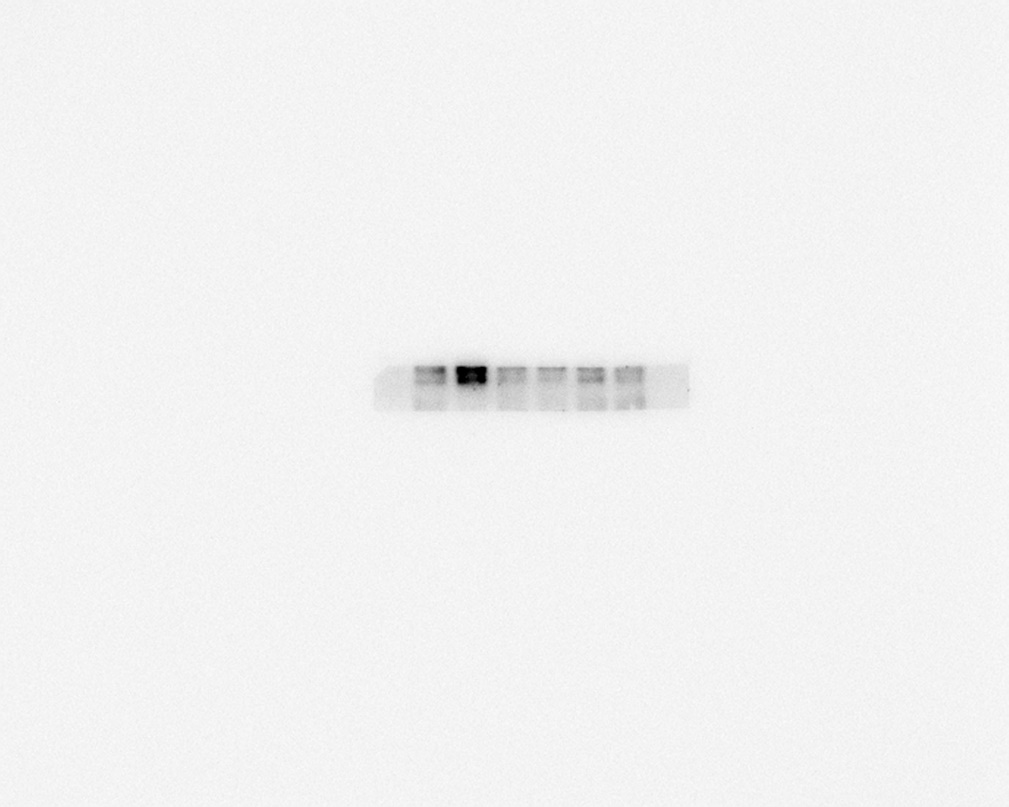

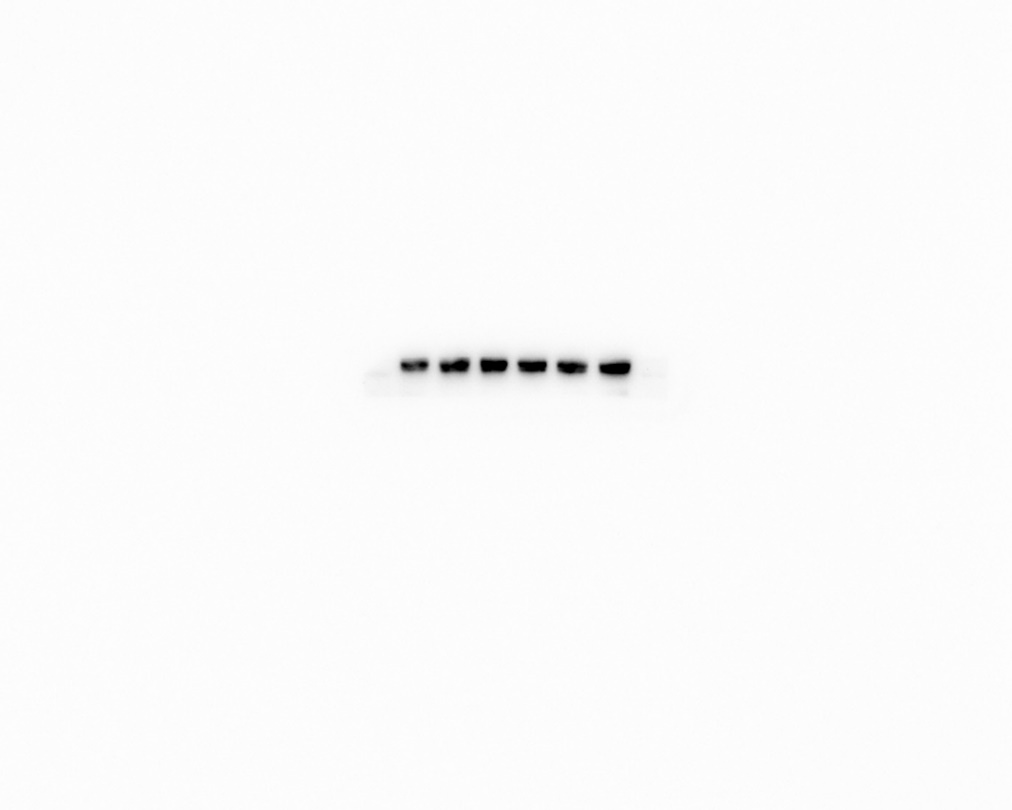

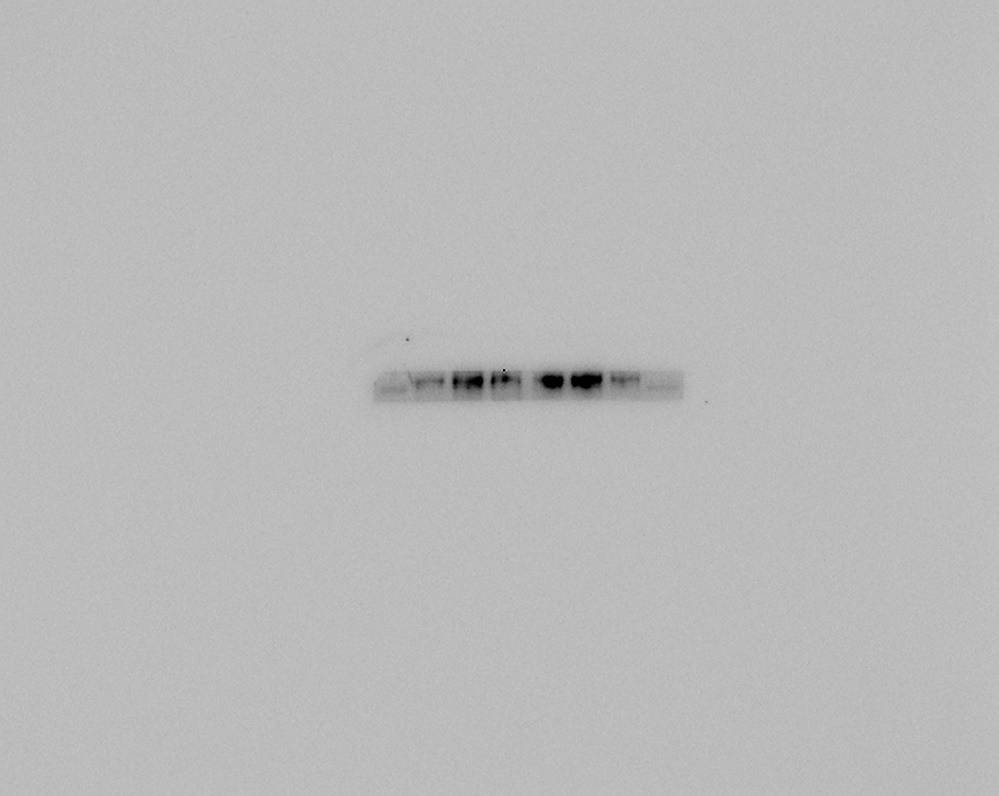

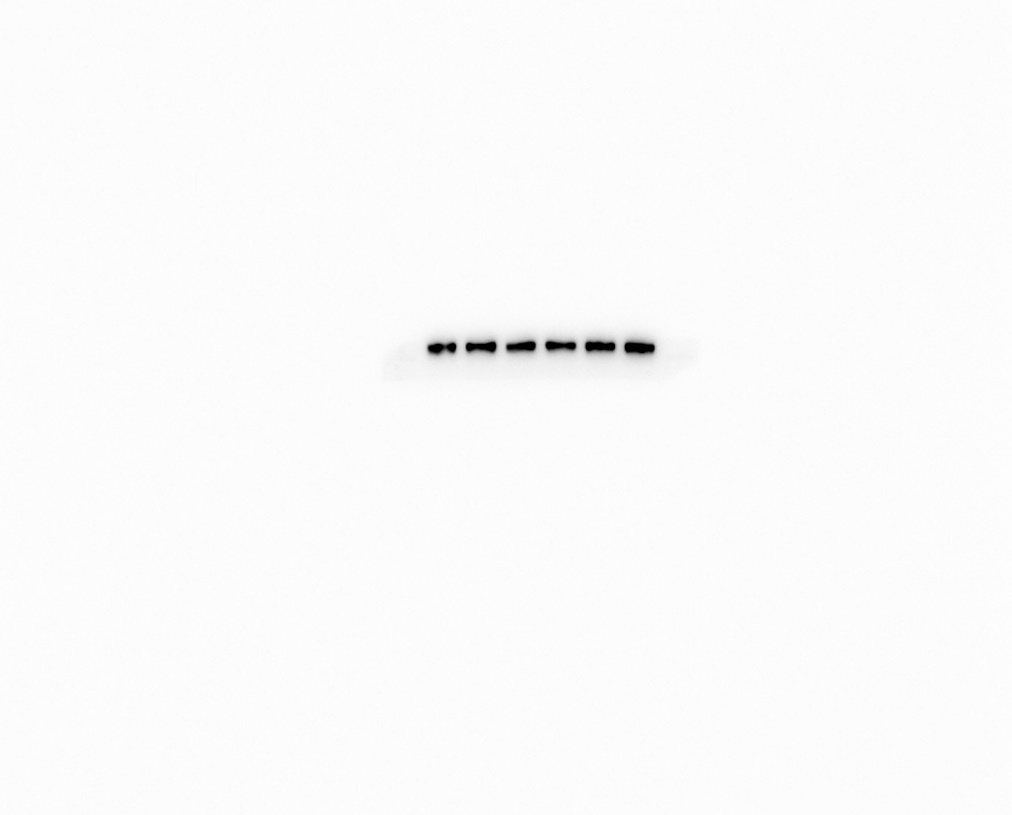

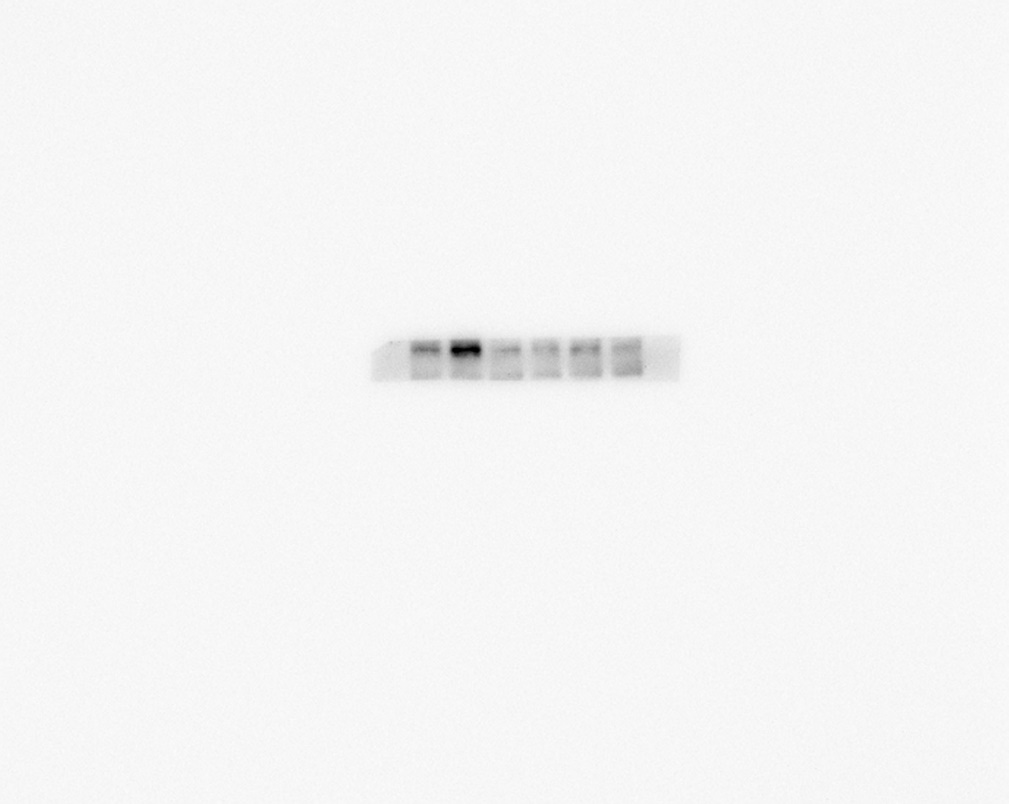

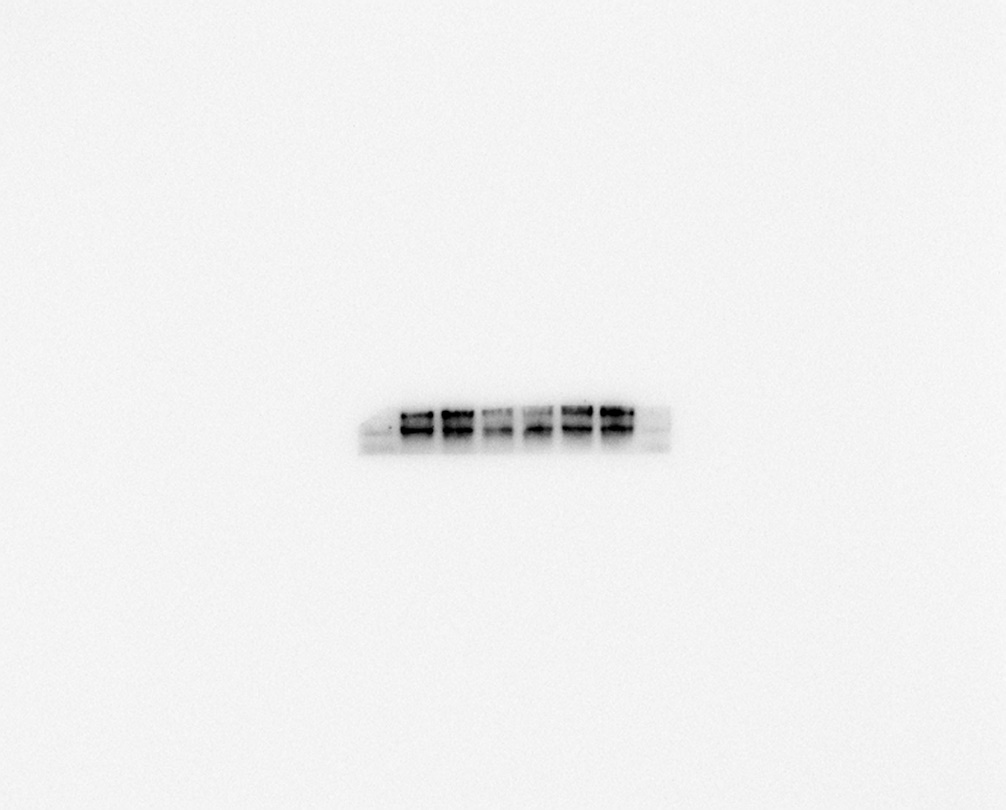


**Blank**

**SADBE**

**0.625**

**1.25**

**2.5**

**Dip**

**QP**

Tubulin

**Blank**

**SADBE**

**0.625**

**1.25**

**2.5**

**Dip**

**QP**

**Blank**

**SADBE**

**0.625**

**1.25**

**2.5**

**Dip**

**QP**

**Blank**

**SADBE**

**0.625**

**1.25**

**2.5**

**Dip**

**QP**

IκBα

P38

p-IκBα

**Blank**

**SADBE**

**0.625**

**1.25**

**2.5**

**Dip**

**QP**

**Blank**

**SADBE**

**0.625**

**1.25**

**2.5**

**Dip**

**QP**

p-Erk

**Blank**

**SADBE**

**0.625**

**1.25**

**2.5**

**Dip**

**QP**

Akt

**Blank**

**SADBE**

**0.625**

**1.25**

**2.5**

**Dip**

**QP**

p-Akt

**Blank**

**SADBE**

**0.625**

**1.25**

**2.5**

**Dip**

**QP**

Stat3

p-P38


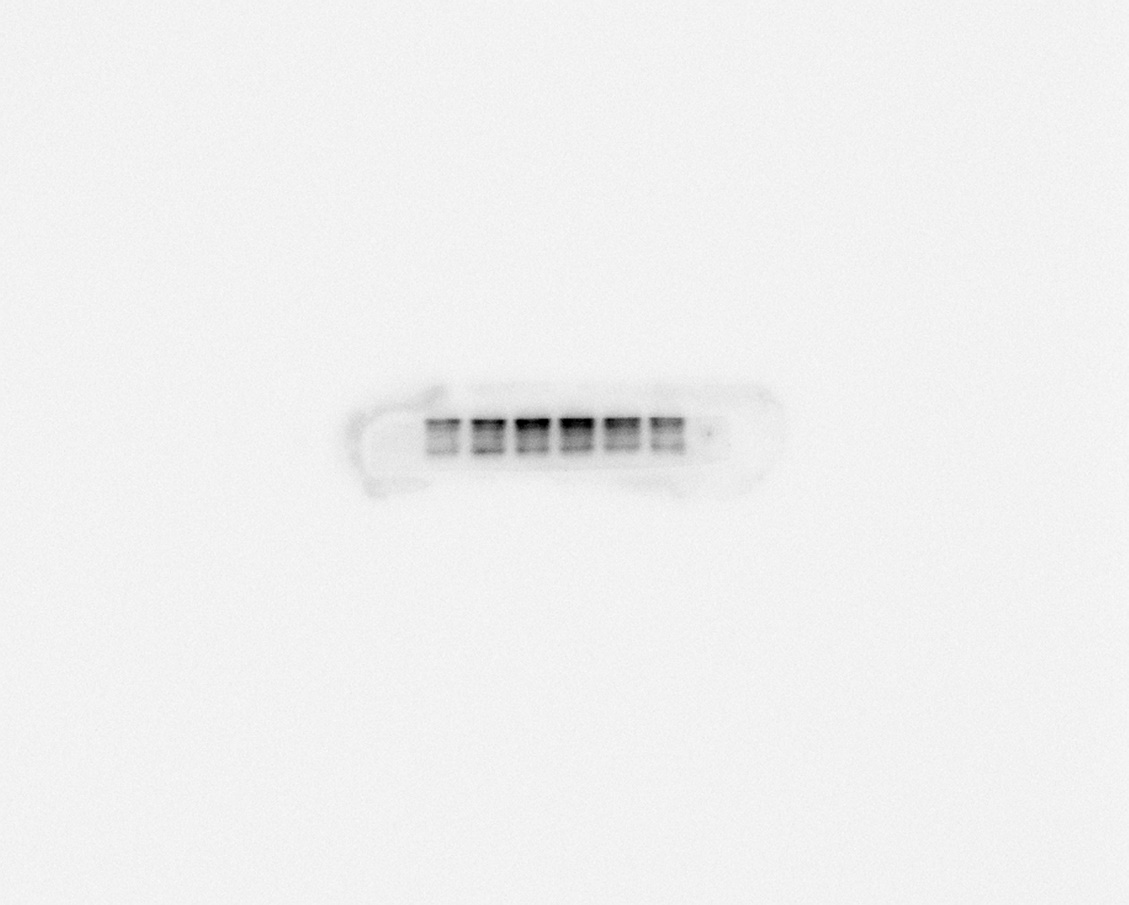


Erk

**Figure S3∣**The original western blot images of **Figure 6A** in the manuscript.

**Blank**

**SADBE**

**0.625**

**1.25**

**2.5**

**Dip**

**QP**

**Blank**

**SADBE**

**0.625**

**1.25**

**2.5**

**Dip**

**QP**


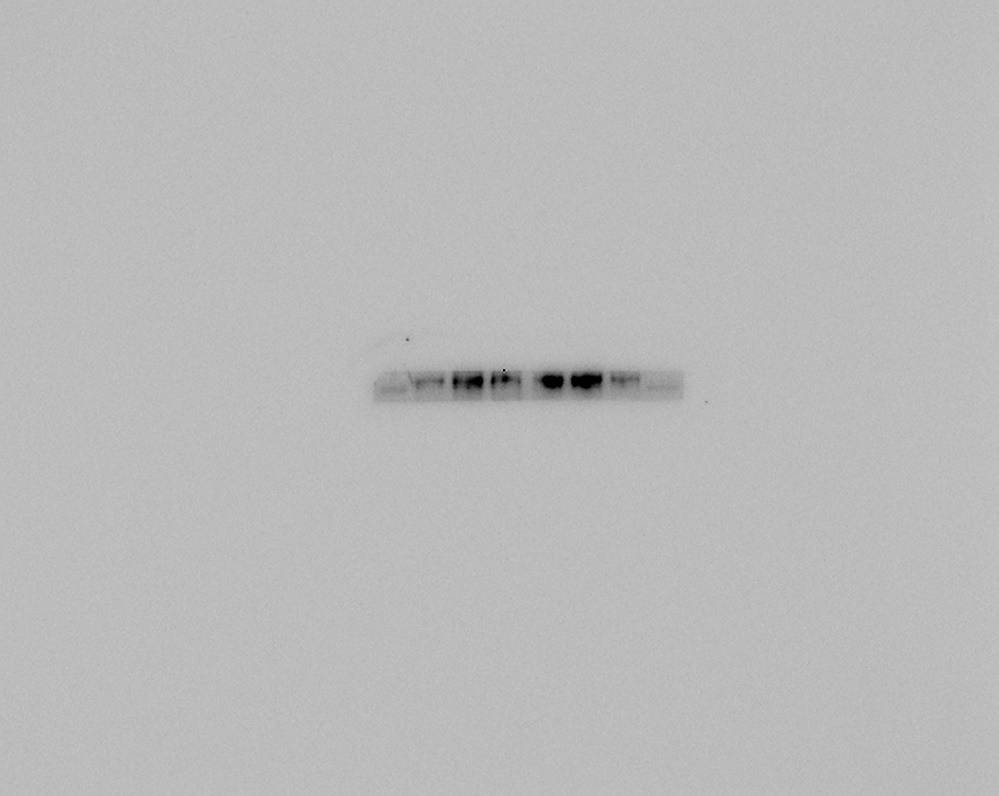

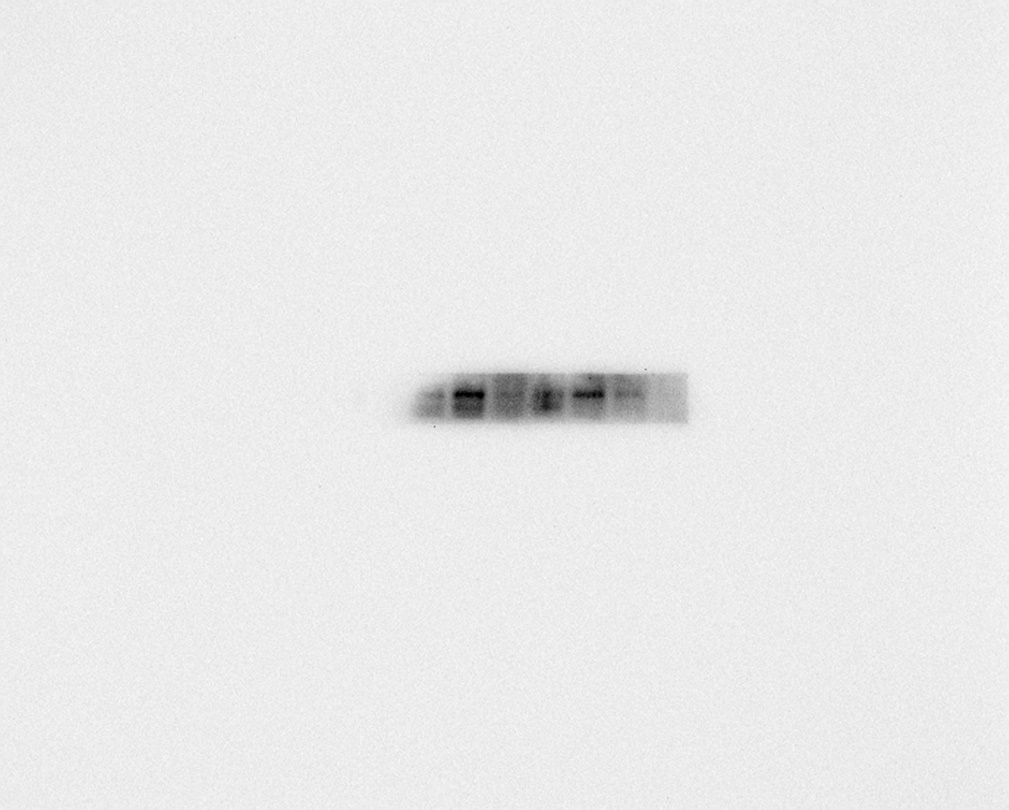


p-IκBα


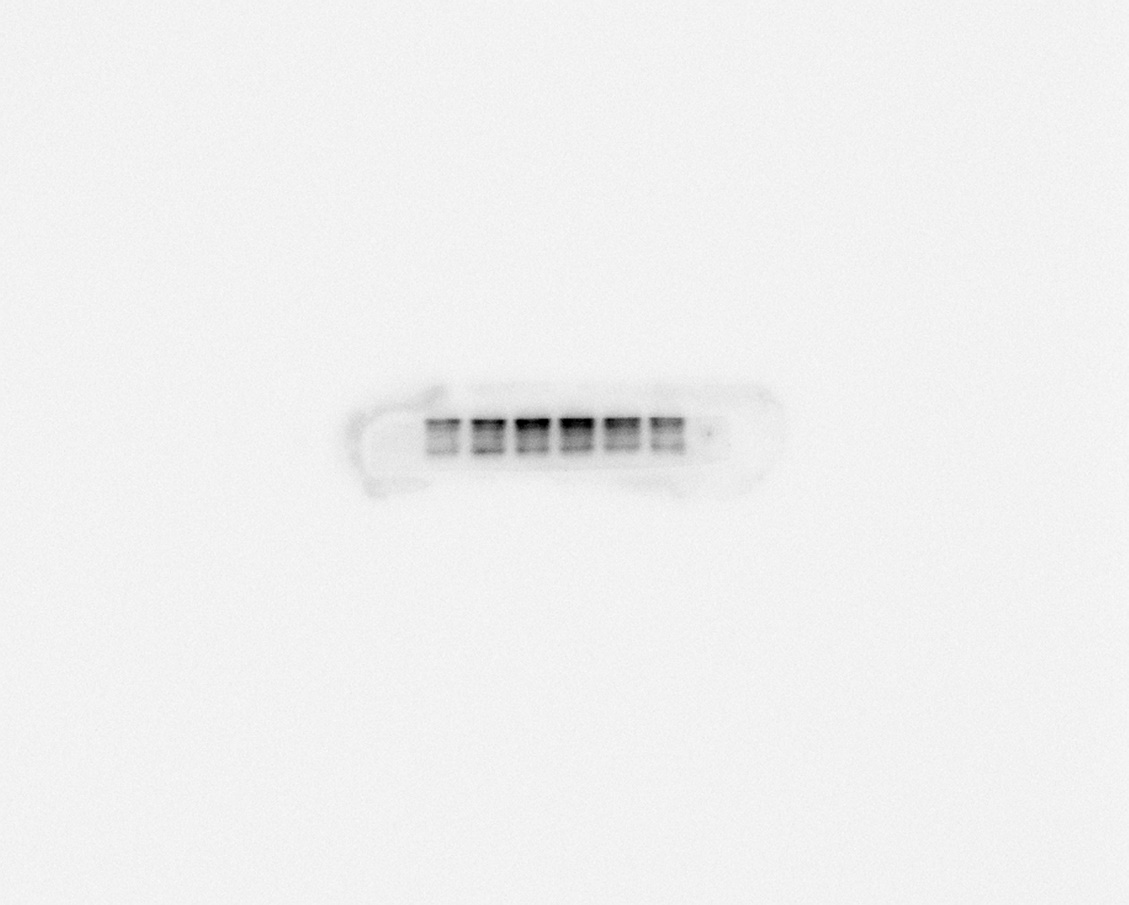

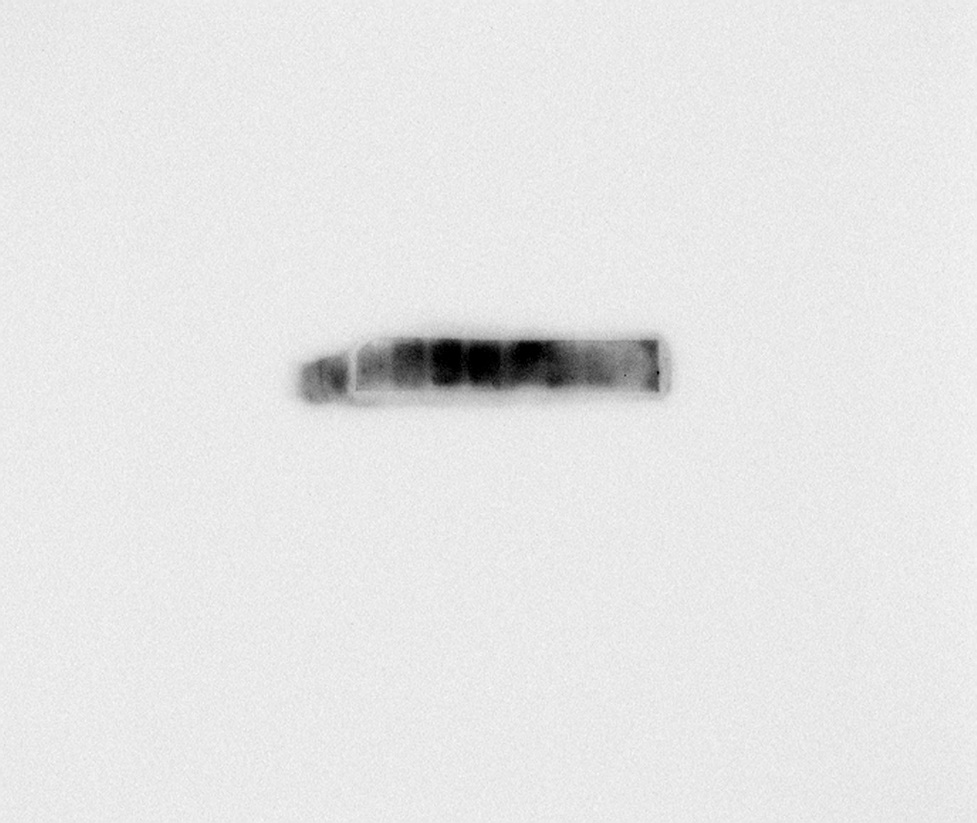

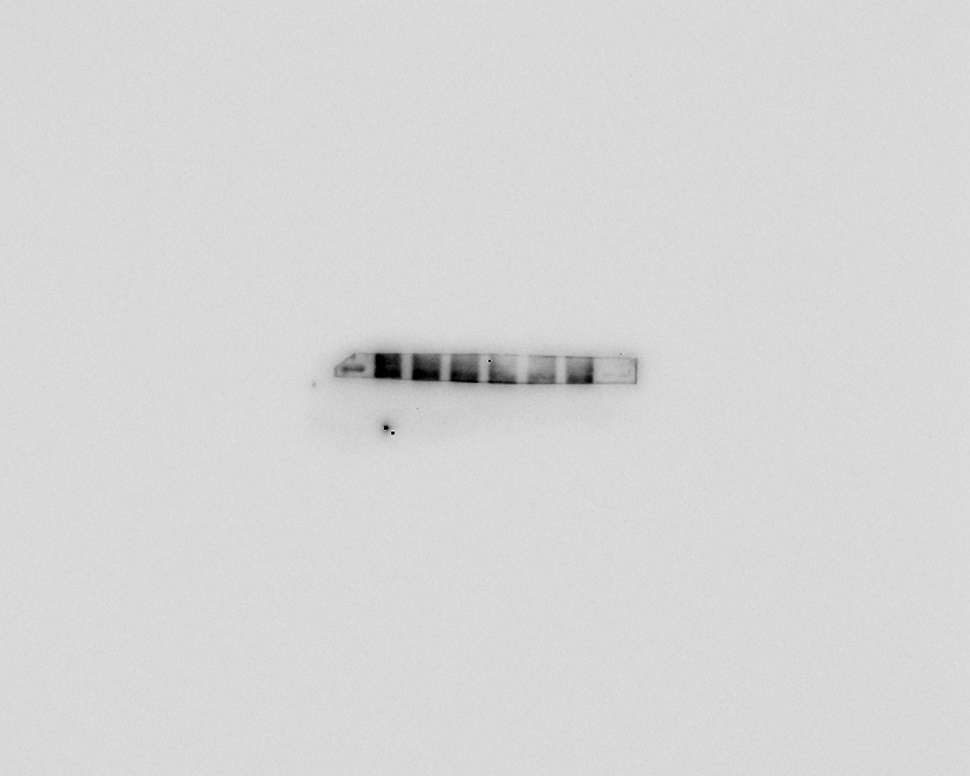

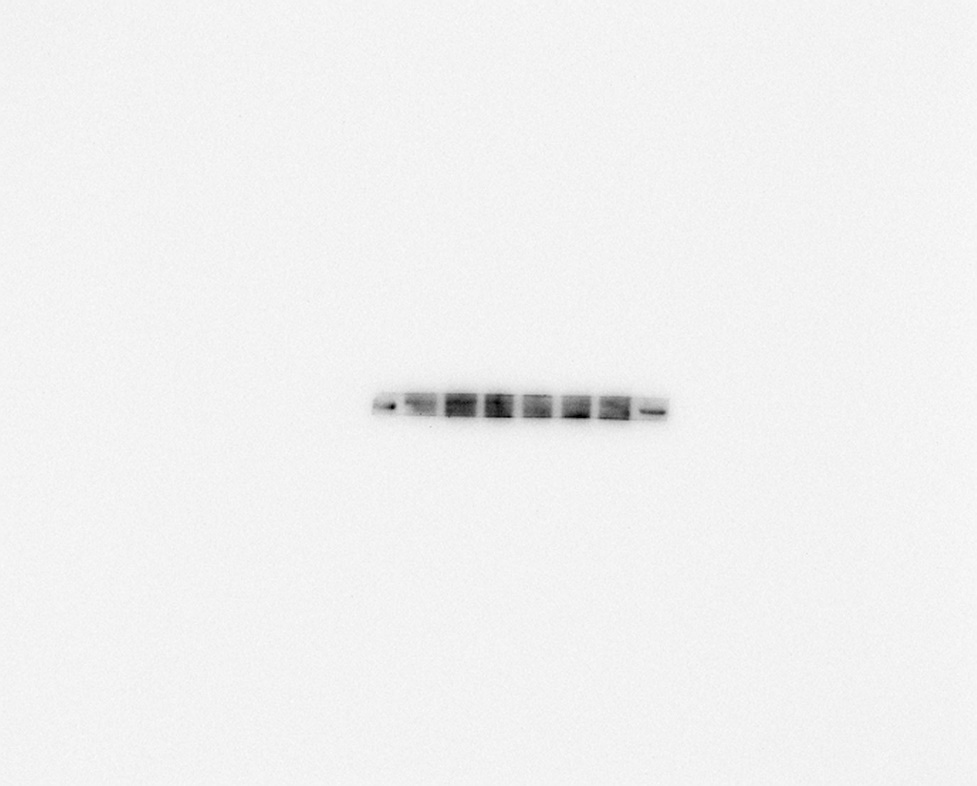


p-Akt

**Blank**

**SADBE**

**0.625**

**1.25**

**2.5**

**Dip**

**QP**

p-Erk


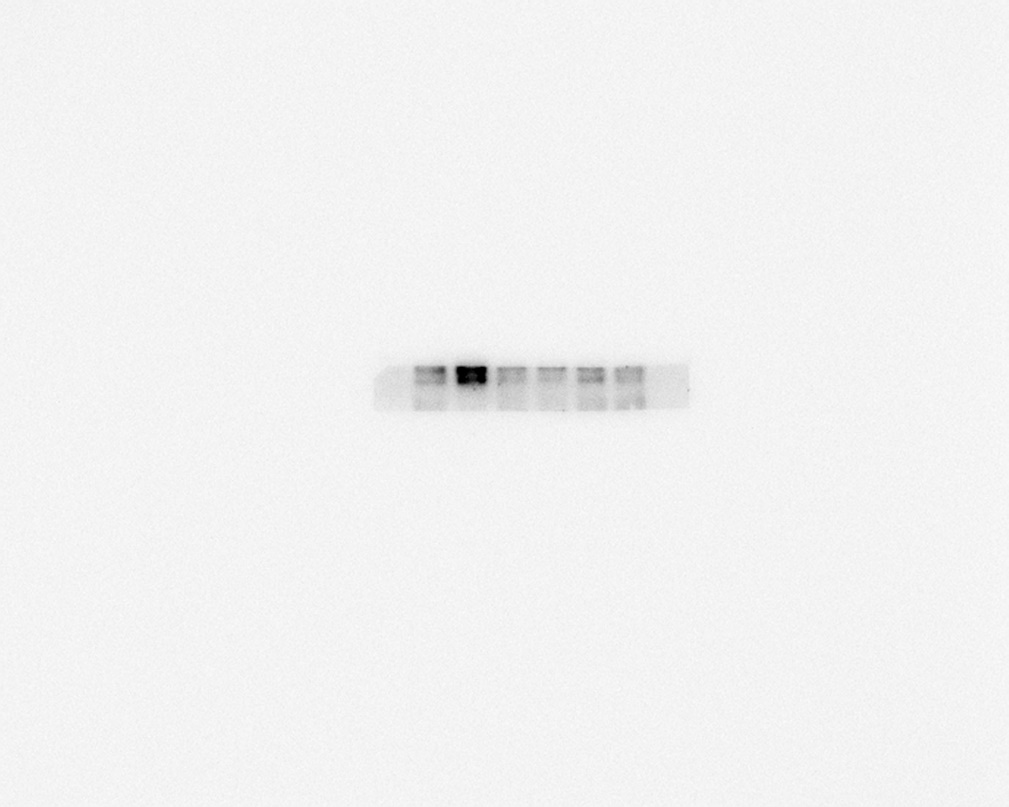


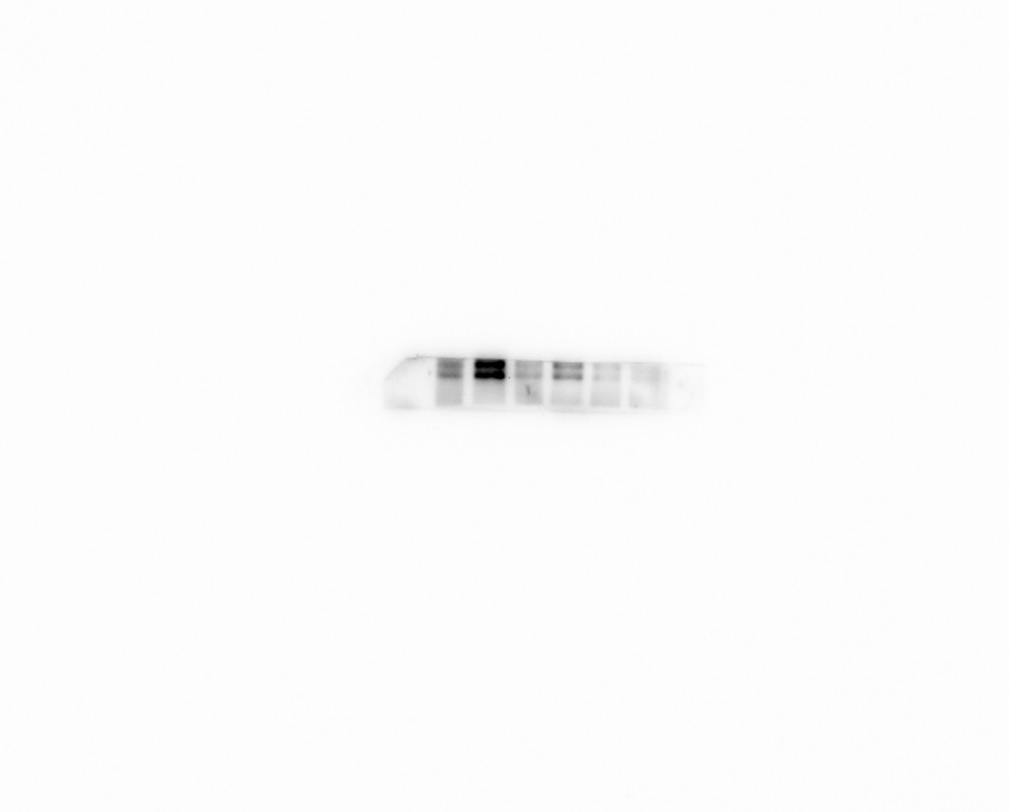


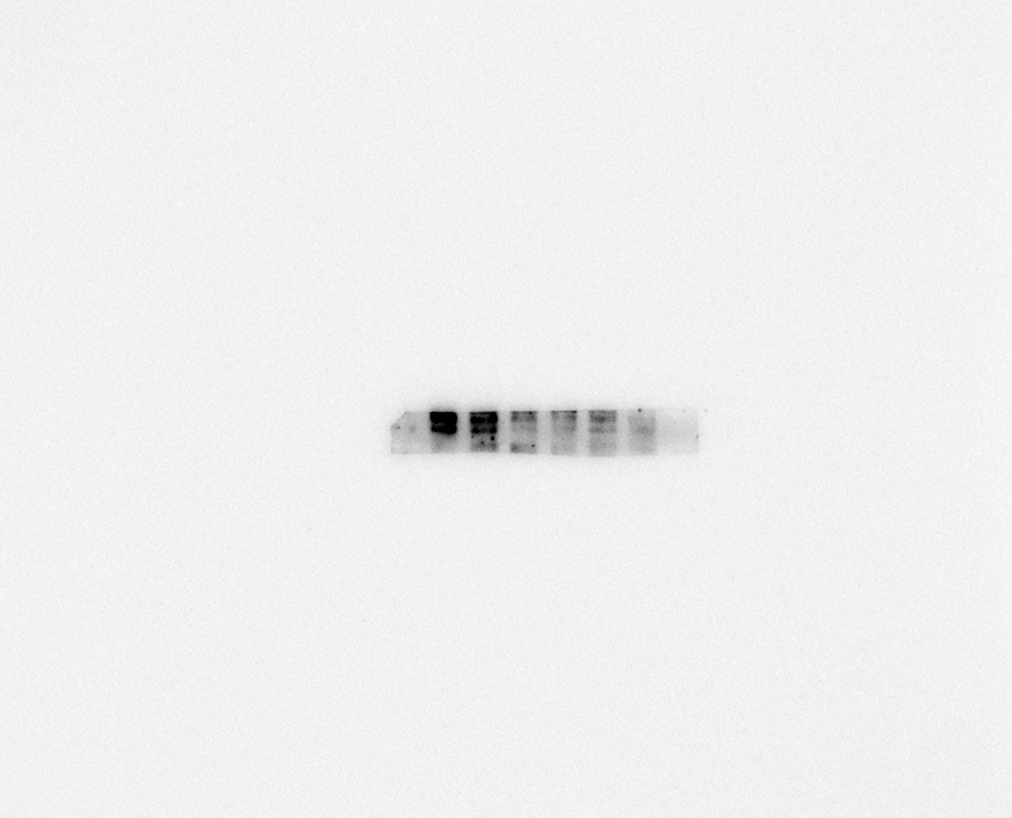


**Figure S4∣**The original western blot images of p-Akt, p-IκBα and p-Erk.

**Blank**

**SADBE**

**0.625**

**1.25**

**2.5**

**Dip**

**QP**

**Blank**

**SADBE**

**0.625**

**1.25**

**2.5**

**Dip**

**QP**


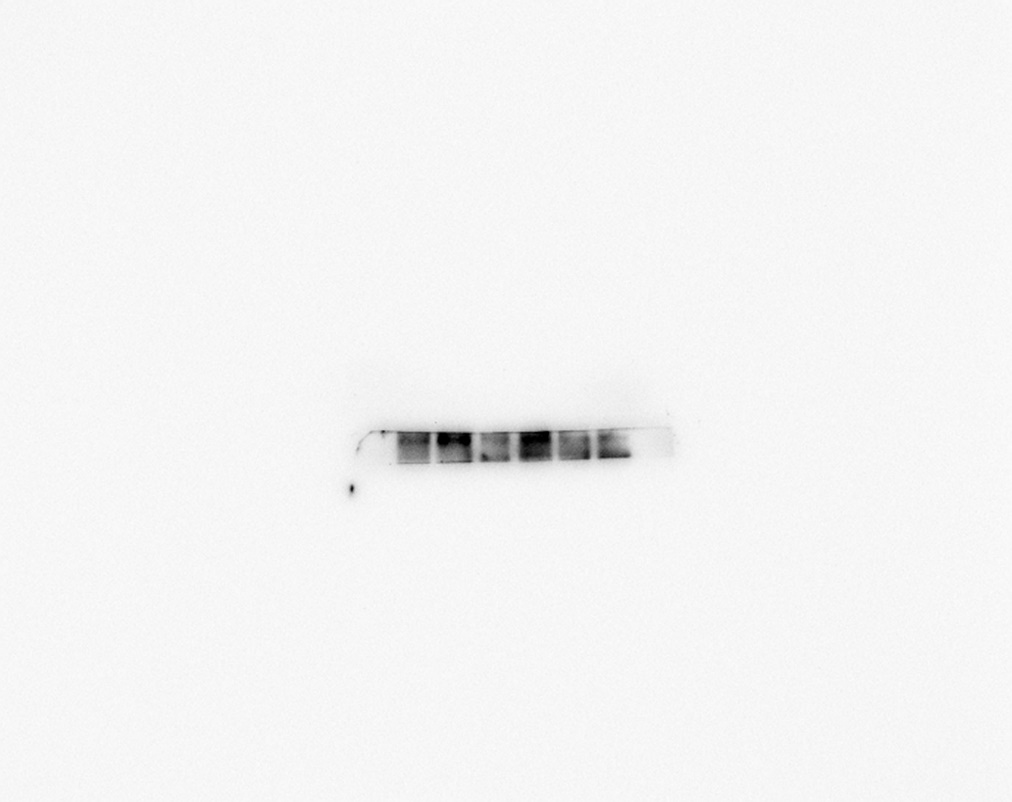

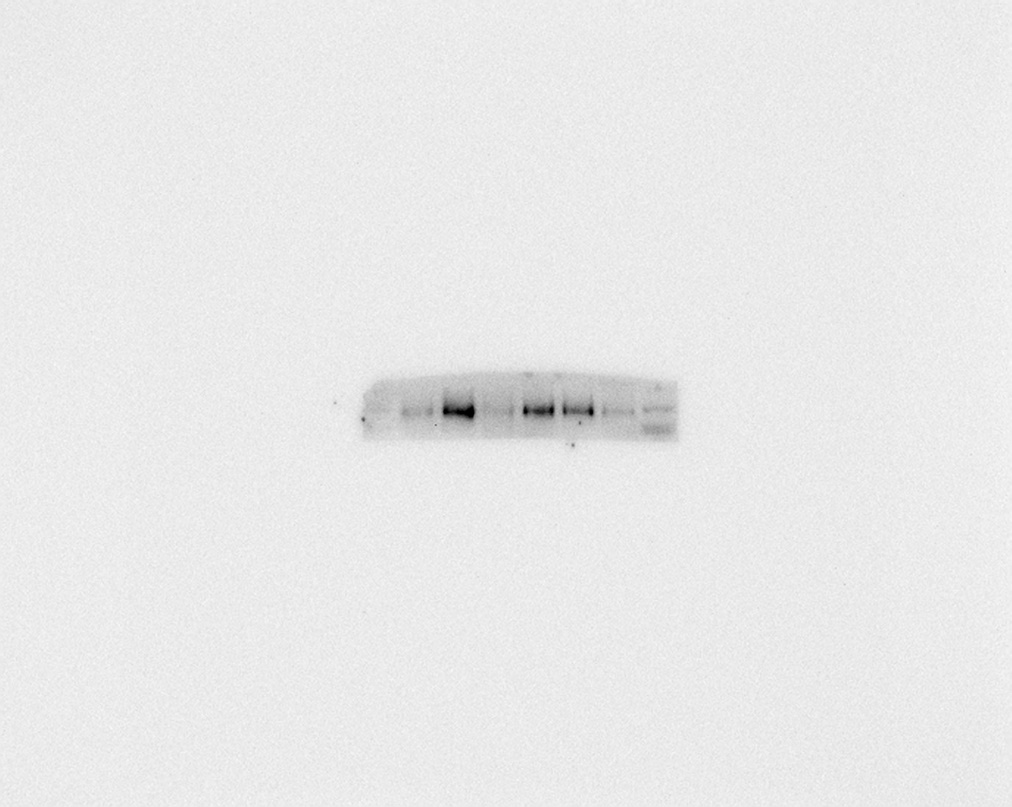


p-Stat3

p-P38

**Blank**

**SADBE**

**0.625**

**1.25**

**2.5**

**Dip**

**QP**

**Blank**

**SADBE**

**0.625**

**1.25**

**2.5**

**Dip**

**QP**


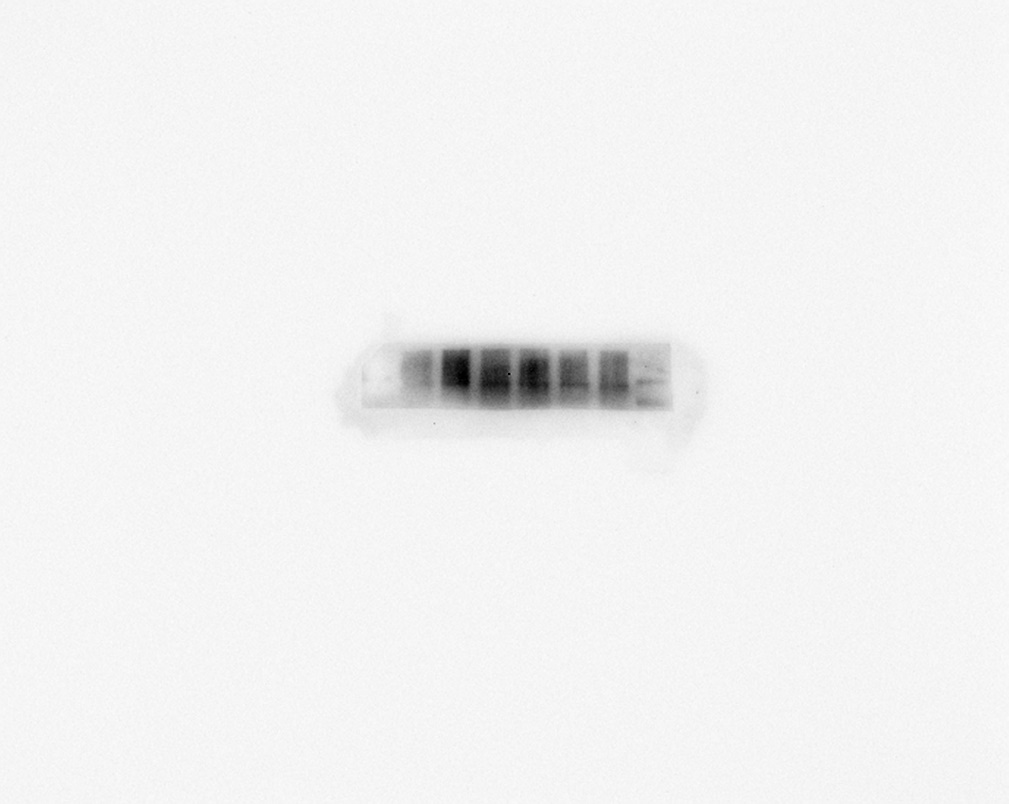


Stat3


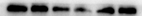


**Blank**

**SADBE**

**0.625**

**1.25**

**2.5**

**Dip**

**QP**

P38

p-Akt


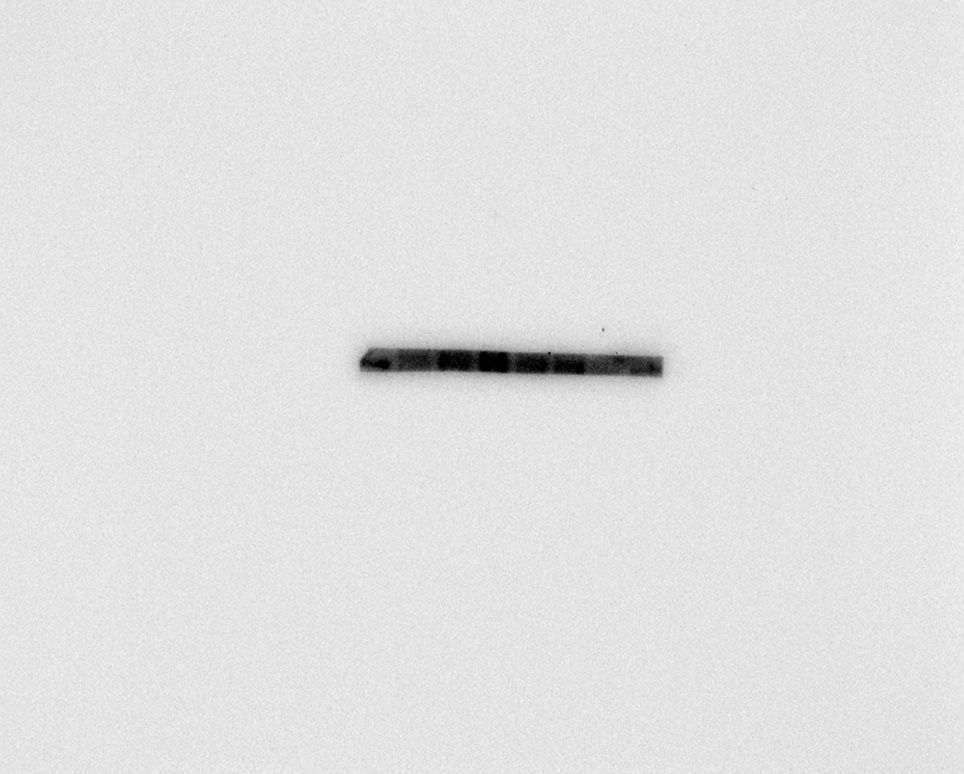


**Blank**

**SADBE**

**0.625**

**1.25**

**2.5**

**Dip**

**QP**

**Blank**

**SADBE**

**0.625**

**1.25**

**2.5**

**Dip**

**QP**


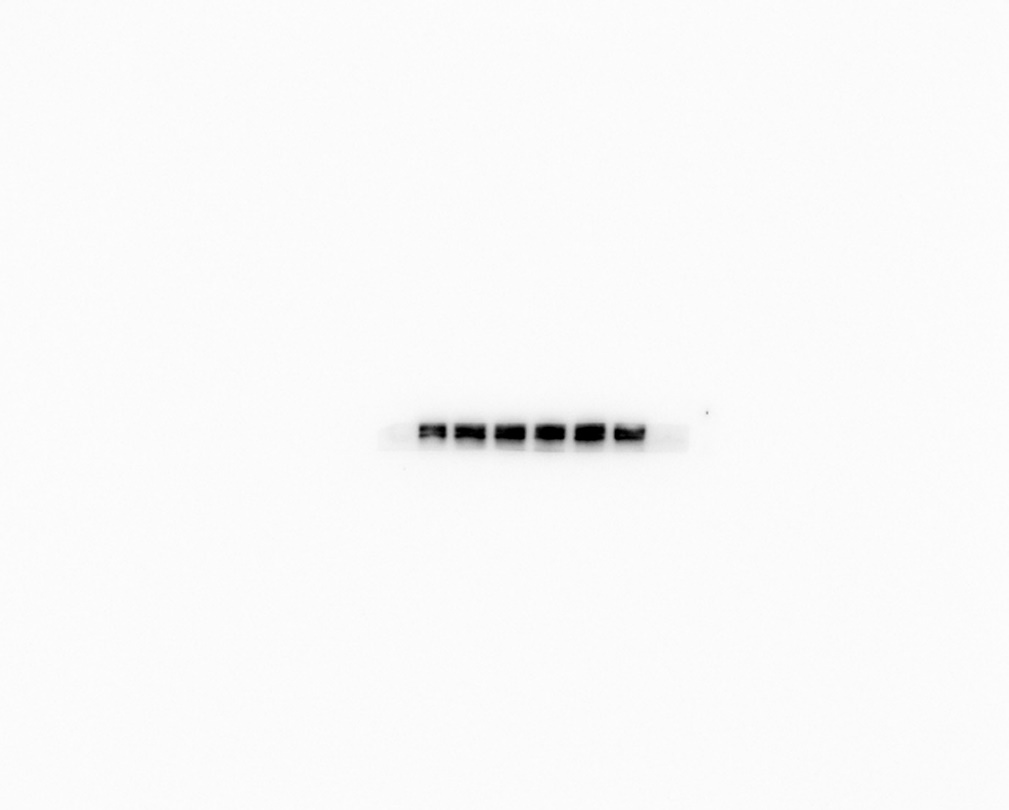

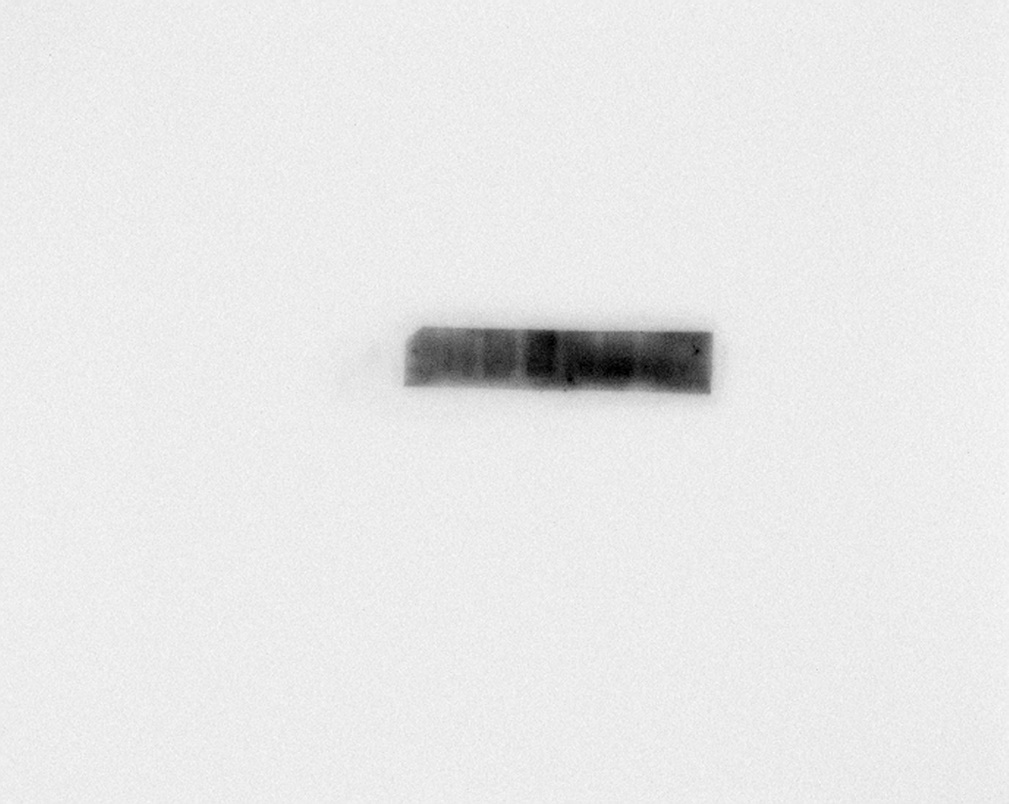


Akt

p-IκBα

**Blank**

**SADBE**

**0.625**

**1.25**

**2.5**

**Dip**

**QP**

**Blank**

**SADBE**

**0.625**

**1.25**

**2.5**

**Dip**

**QP**


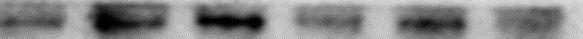

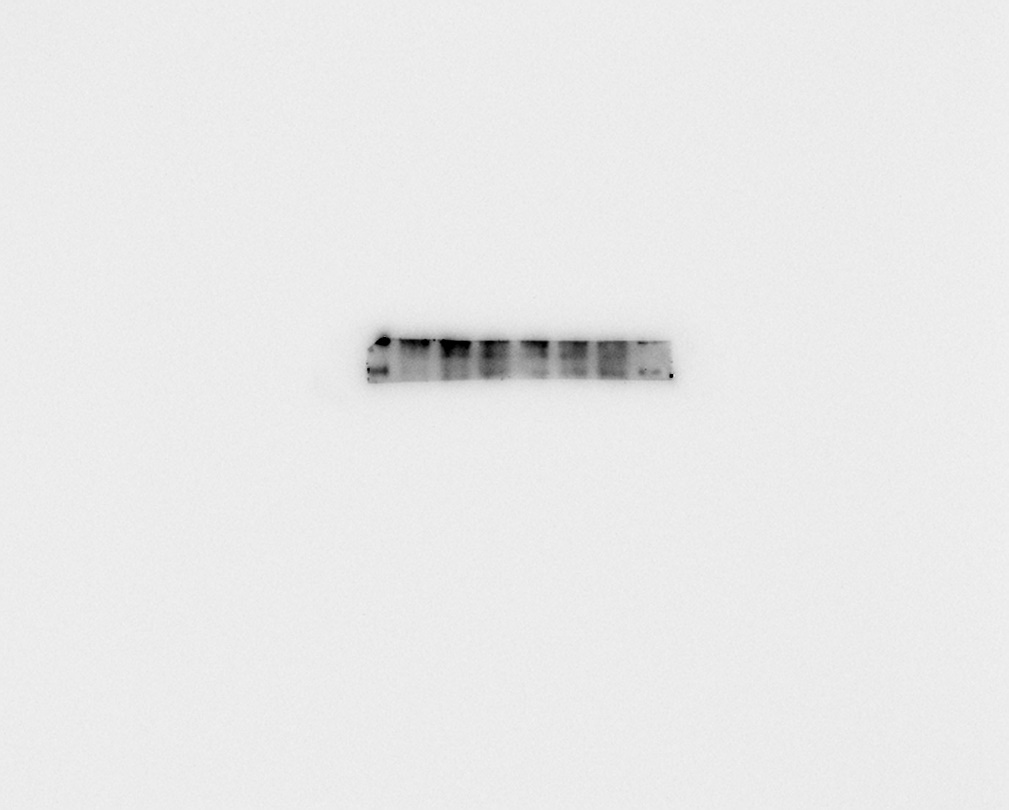


p-Erk

IκBα


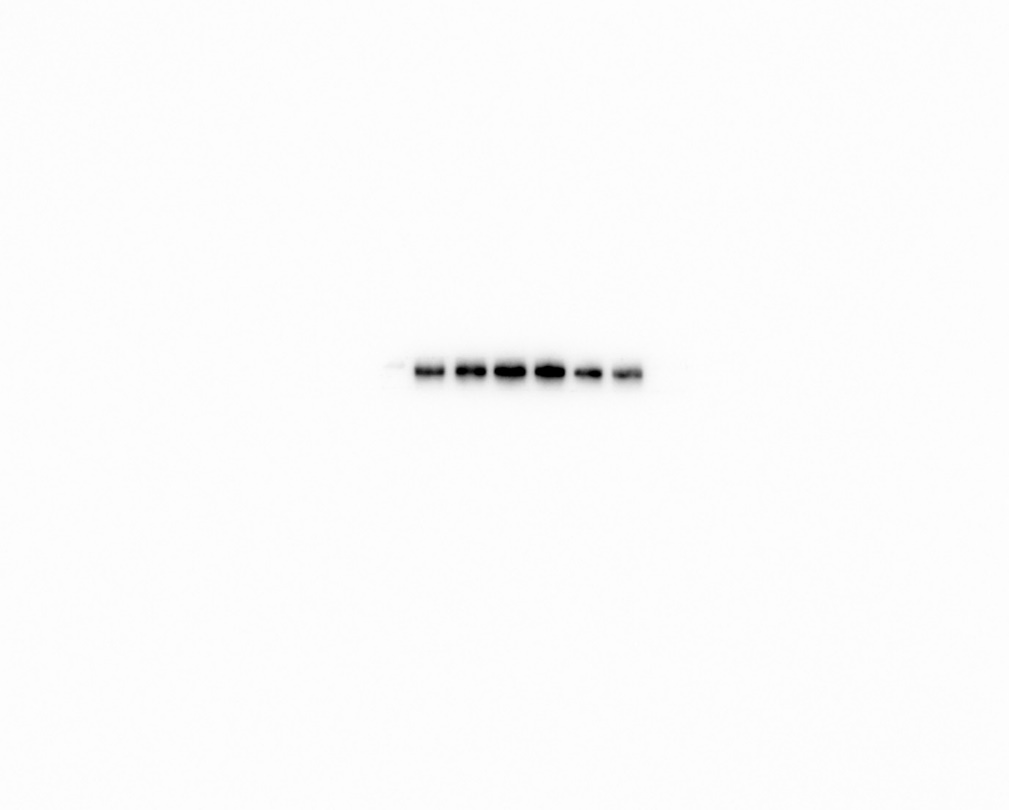

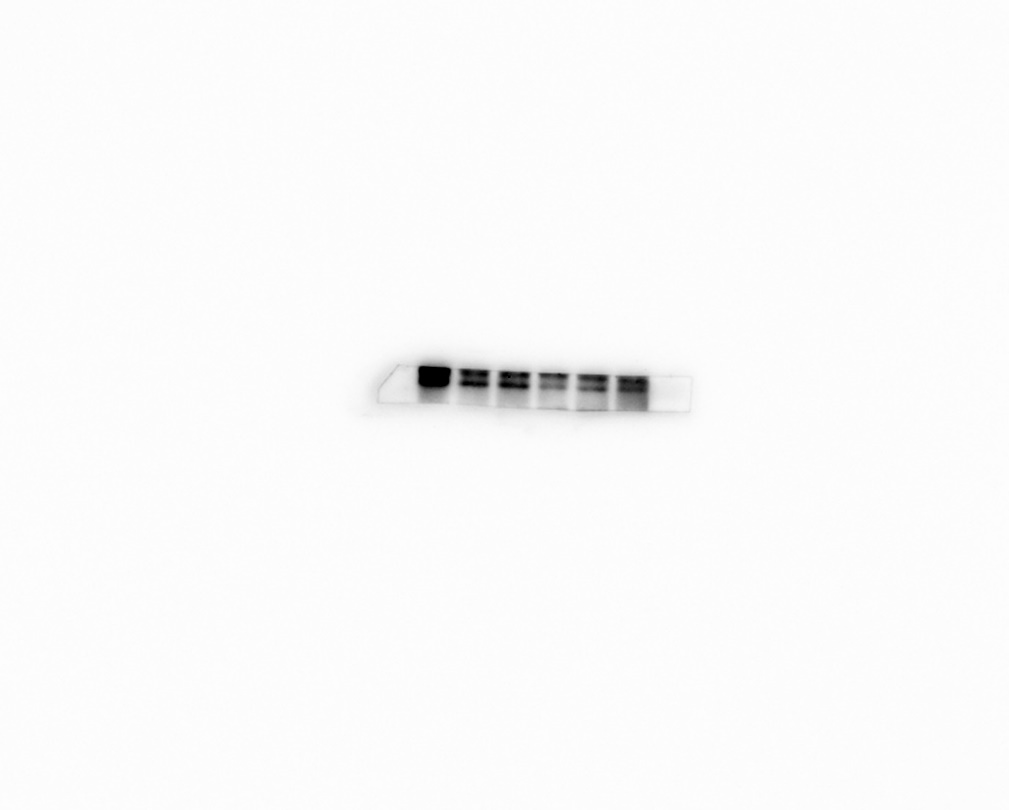


**Figure S5∣**The original western blot images of **Figure S2** in the manuscript.

**Blank**

**SADBE**

**0.625**

**1.25**

**2.5**

**Dip**

**QP**

**Blank**

**SADBE**

**0.625**

**1.25**

**2.5**

**Dip**

**QP**

Tubulin

Erk
